# Multi-Threshold Image Segmentation Based on the Hybrid Strategy Improved Dingo Optimization Algorithm

**DOI:** 10.3390/biomimetics11010052

**Published:** 2026-01-08

**Authors:** Qianqian Zhu, Min Gong, Yijie Wang, Zhengxing Yang

**Affiliations:** College of Design, Hanyang University, Ansan 15588, Gyeonggi-do, Republic of Korea; zhuqianqian@hanyang.ac.kr (Q.Z.); gm510@hanyang.ac.kr (M.G.); sunysen@hanyang.ac.kr (Y.W.)

**Keywords:** Dingo Optimization Algorithm, multi-threshold image segmentation, quadratic interpolation search, horizontal crossover, global optimization

## Abstract

This study proposes a Hybrid Strategy Improved Dingo Optimization Algorithm (HSIDOA), designed to address the limitations of the standard DOA in complex optimization tasks, including its tendency to fall into local optima, slow convergence speed, and inefficient boundary search. The HSIDOA integrates a quadratic interpolation search strategy, a horizontal crossover search strategy, and a centroid-based opposition learning boundary-handling mechanism. By enhancing local exploitation, global exploration, and out-of-bounds correction, the algorithm forms an optimization framework that excels in convergence accuracy, speed, and stability. On the CEC2017 (30-dimensional) and CEC2022 (10/20-dimensional) benchmark suites, the HSIDOA achieves significantly superior performance in terms of average fitness, standard deviation, convergence rate, and Friedman test rankings, outperforming seven mainstream algorithms including MLPSO, MELGWO, MHWOA, ALA, HO, RIME, and DOA. The results demonstrate strong robustness and scalability across different dimensional settings. Furthermore, HSIDOA is applied to multi-level threshold image segmentation, where Otsu’s maximum between-class variance is used as the objective function, and PSNR, SSIM, and FSIM serve as evaluation metrics. Experimental results show that HSIDOA consistently achieves the best segmentation quality across four threshold levels (4, 6, 8, and 10 levels). Its convergence curves exhibit rapid decline and early stabilization, with stability surpassing all comparison algorithms. In summary, HSIDOA delivers comprehensive improvements in global exploration capability, local exploitation precision, convergence speed, and high-dimensional robustness. It provides an efficient, stable, and versatile optimization method suitable for both complex numerical optimization and image segmentation tasks.

## 1. Introduction

In the rapid development of artificial intelligence, computer vision, and engineering optimization, the scale and complexity of optimization problems continue to grow. Traditional deterministic optimization methods—due to their reliance on gradient information and their tendency to become trapped in local optima—are increasingly unable to meet the demands of high-dimensional, nonlinear, and multimodal problem settings [1,2]. As stochastic optimization methods inspired by natural phenomena or biological behaviors, metaheuristic algorithms have emerged as essential tools for solving complex optimization problems. Owing to their gradient-free nature, strong global search capability, and high robustness [3,4], they have been widely applied to numerical optimization, image segmentation [5], path planning [6], neural network parameter tuning [7], resource scheduling [8], and many other real-world domains.

Multi-level threshold image segmentation, as a fundamental task in computer vision, classifies image pixels into multiple categories by setting several gray-level thresholds. This approach preserves structural characteristics and fine details of the image more comprehensively, making it indispensable in critical applications such as medical image analysis, remote sensing image processing, and industrial defect detection [9,10]. However, the core challenge of multi-threshold image segmentation lies in determining the optimal combination of thresholds. The objective functions used in this process—such as maximum between-class variance and entropy—typically exhibit high dimensionality and strong multimodality. Traditional methods based on enumeration or iterative search suffer from low computational efficiency, underscoring the need for high-performance optimization algorithms [11,12]. Meanwhile, increasing image resolution and texture complexity place higher demands on segmentation accuracy, convergence speed, and algorithmic stability. Balancing global exploration and local exploitation thus becomes a key challenge for enhancing the performance of multi-level threshold image segmentation.

Existing metaheuristic algorithms still exhibit several common limitations in numerical optimization and image segmentation tasks. Some algorithms experience rapid loss of population diversity in the later stages of iteration, making them prone to premature convergence and local optima [13,14]. Others suffer from insufficient local exploitation capability, preventing them from effectively refining the neighborhood of the optimal solution. In addition, certain algorithms adopt overly simplistic boundary-handling mechanisms for out-of-bounds solutions, which may lead to the loss of valuable information and reduce search efficiency in boundary regions [7,15]. Therefore, developing a novel metaheuristic algorithm that simultaneously balances global exploration and local exploitation, ensures both convergence accuracy and speed, and maintains stability and adaptability holds significant theoretical importance and practical value for solving complex optimization problems efficiently.

Since the concept of metaheuristic algorithms was introduced, hundreds of optimization methods inspired by various natural phenomena have been proposed by researchers worldwide, forming a rich algorithmic ecosystem. Among the early classical algorithms, Particle Swarm Optimization (PSO) simulates the foraging behavior of bird flocks, where individuals share information with the group to guide the search. However, PSO often suffers from slow convergence in later iterations and is prone to being trapped in local optima [16]. The Grey Wolf Optimizer (GWO), inspired by the encircling, hunting, and attacking behaviors of grey wolves, performs well in low-dimensional optimization but experiences a significant decline in search efficiency as dimensionality increases [17,18]. The Whale Optimization Algorithm (WOA), based on the bubble-net foraging strategy of humpback whales, offers strong global exploration capability but exhibits limited local exploitation performance [19,20].

According to the No Free Lunch (NFL) theorem [21], no single optimization algorithm can achieve superior performance over all possible optimization problems. This theorem implies that an algorithm that performs well on a specific class of problems may exhibit inferior performance on other problem types. Consequently, designing problem-oriented or hybrid optimization algorithms has become a widely accepted and effective strategy to improve optimization performance in practical applications. To overcome the limitations of classical algorithms, researchers have proposed various improvements through hybrid strategies, parameter adaptation, and enhanced local search mechanisms. For example, the Multi-Layer Particle Swarm Optimization algorithm (MLPSO) adopts a hierarchical clustering mechanism to increase population diversity, thereby improving performance on multimodal problems [22]. The Memory Evolutionary Operator and Local Search-based GWO (MELGWO) incorporates memory mechanisms and stochastic local search, significantly strengthening local exploitation capability [23]. The Multi-Strategy Hybrid Whale Optimization Algorithm (MHWOA) integrates multiple search strategies to achieve a better balance between global exploration and local exploitation [24]. Although these enhanced algorithms improve optimization performance in specific scenarios, they still present several limitations. Some methods focus on mitigating a single performance deficiency and struggle to address multidimensional performance requirements comprehensively; others introduce overly complex strategies, resulting in substantially increased computational overhead and reduced practical efficiency; additionally, certain algorithms exhibit weak adaptability, leading to unstable performance across problems of different dimensionalities and characteristics [25,26,27]. Nevertheless, despite their successes across various applications, traditional intelligent optimization methods still suffer from several inherent drawbacks, such as susceptibility to local optima, limited convergence precision, and insufficient information-sharing mechanisms within the population. These issues further weaken their capability to explore the solution space effectively.

The Dingo Optimization Algorithm (DOA), introduced in 2021, is a novel metaheuristic algorithm inspired by the group attack, pursuit, scavenging, and survival behaviors of Australian dingoes. It establishes a balanced framework between global exploration and local exploitation [28]. Although the DOA performs well in its initial studies, it exhibits noticeable limitations when addressing high-dimensional and complex optimization problems: its scavenging behavior generates new solutions in a simplistic manner, lacking the ability to exploit nonlinear interactions among high-quality solutions; population updating relies heavily on individual behaviors with insufficient information exchange, leading to rapid loss of diversity and a tendency to fall into local optima; and its straightforward boundary-handling mechanism often discards useful information and reduces search efficiency [29,30]. These shortcomings restrict the applicability of the DOA in real-world multi-threshold image segmentation tasks, highlighting the need for hybrid strategies to enhance its overall performance.

To address the weaknesses of the DOA—including inadequate local exploitation, rapid decline in population diversity, and low boundary search efficiency—this study proposes a Hybrid Strategy Improved Dingo Optimization Algorithm (HSIDOA). The algorithm incorporates three key mechanisms for systematic enhancement. First, a quadratic interpolation search strategy is employed to construct an interpolation model among high-quality solutions, enabling deeper exploration of the nonlinear structure of the solution space and thus strengthening local exploitation. Second, a horizontal crossover search strategy is introduced to facilitate information exchange among individuals at the population level, effectively maintaining diversity and improving global exploration capability. Finally, a centroid-based opposition learning boundary-handling strategy is designed, which reconstructs out-of-bounds solutions via reflection around the population centroid rather than simple truncation, thereby preserving valuable information and significantly improving search efficiency near the boundaries. Through these multidimensional hybrid improvements, HSIDOA aims to comprehensively enhance the performance of DOA when addressing high-dimensional and complex optimization problems.

The main contributions of this study are as follows:

(1) A hybrid improved Dingo Optimization Algorithm (HSIDOA) integrating three enhancement strategies is proposed to address the core limitations of the standard DOA, including weak local exploitation, rapid loss of population diversity, and inefficient boundary handling. This provides a new solution framework for tackling complex optimization problems.
(2) Extensive experiments on the CEC2017 and CEC2022 benchmark suites are conducted to comprehensively validate the superiority of HSIDOA.
(3) HSIDOA is successfully applied to multi-threshold image segmentation tasks, demonstrating its practical effectiveness in real engineering applications and offering new technical support for advancing image segmentation methods.

The remainder of this paper is organized as follows: Section 2 introduces the fundamental principles of DOA and presents the three enhancement strategies as well as the overall framework of the proposed HSIDOA. Section 3 compares HSIDOA with several state-of-the-art optimization algorithms on the CEC benchmark tests. Section 4 applies HSIDOA to multi-threshold image segmentation and evaluates its practical performance. Section 5 concludes the study and discusses potential directions for future research.

## 2. Dingo Optimization Algorithm and the Proposed HSIDOA

### 2.1. Dingo Optimization Algorithm (DOA)

**Figure 1:**
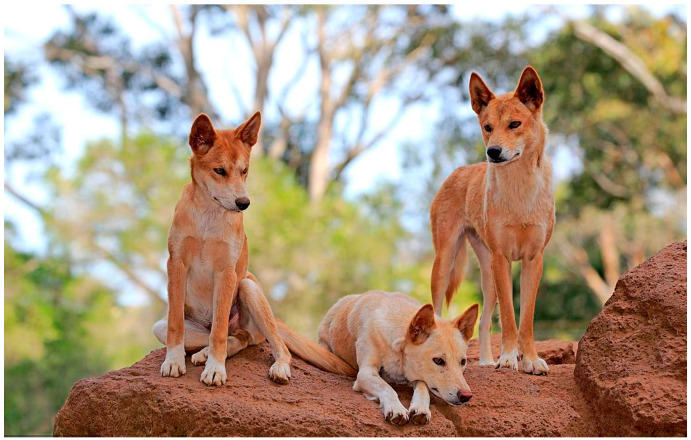
The appropriate image of ‘Dingo’.

The Dingo Optimization Algorithm (DOA) is a novel metaheuristic algorithm proposed in 2021, inspired by the social behaviors of Australian dingoes. Figure 1 Illustration of Australian dingo, which inspires the Dingo Optimization Algorithm (DOA). The algorithm simulates their hunting strategies—such as persecution attacks, group-based coordination, and scavenging behavior—to achieve a balance between global exploration and local exploitation. Its mathematical formulation is as follows [28,29]:

**Strategy 1: Group Attack**

Australian dingoes typically hunt in groups. They can identify the prey’s position and coordinate to encircle it. This cooperative attack behavior is modeled by Equation (1) [28,31]:

(1) $$X_{i}\left(t+1\right)=\beta_{1}\sum_{k=1}^{na}\frac{\left[\varphi_{k}(t)-X_{i}(t)\right]}{na}-X_{gbest}(t)$$

where $X_{i}\left(t+1\right)$ denotes the updated position of the search agent (representing the movement of a dingo); i denotes the index of the i-th dingo (search agent) in the population. The subset of search agents refers to a randomly selected group of dingoes participating in the cooperative group attack behavior, rather than the entire population. na is an integer randomly generated within the interval $\left[2,\frac{Pop}{2}\right]$, and Pop is the total population size; $\varphi_{k}(t)$ represents a subset of search agents (i.e., dingoes participating in the attack), where φ⊂X and X denotes the randomly initialized dingo population; $X_{i}(t)$ is the current search agent; $X_{gbest}(t)$ is the best solution found in the previous iteration; $\beta_{1}$ is a scaling factor uniformly sampled from [−2, 2] that controls the magnitude and direction of the dingoes’ movement trajectory [28,29].

**Strategy 2: Pursuit**

Australian dingoes often chase small prey individually until capture. This pursuit behavior is modeled by Equation (2) [28,29]:

(2) $$X_{i}\left(t+1\right)=X_{gbest}(t)+\beta_{1}\cdot e^{\beta_{2}}\cdot (X_{r_{1}}(t)-X_{i}(t))$$

where $\beta_{2}$ is a uniformly generated random number within the interval [−1, 1]; $X_{r_{1}}$ is a randomly selected search agent from the interval [1, Pop], with $i\neq r_{1}$.

**Strategy 3: Scavenging**

Scavenging behavior refers to dingoes discovering and consuming carrion while randomly roaming their habitat. This behavior is modeled by Equation (3) [28,29]:

(3) $$X_{i}\left(t+1\right)=\frac{1}{2}\left[e^{\beta_{2}}\cdot X_{r_{1}}(t)-(-1{)}^{\sigma }\cdot X_{i}(t)\right]$$

where $X_{r_{1}}(t)$ is a randomly selected $r_{1}$-th search agent; $X_{i}(t)$ is the current search agent, with $i\neq r_{1}$; σ is a randomly generated binary number.

**Strategy 4: Survival Rate of Australian Dingoes**

Australian dingoes face the risk of extinction primarily due to illegal hunting. In the Dingo Optimization Algorithm, the survival rate of each dingo is defined by Equation (4) [28,29]:

(4) $$survival(i)=\frac{{\;fitness\;}_{max}-\;fitness\;(i)}{{\;fitness\;}_{max}-{\;fitness\;}_{min}}$$

where ${fitness}_{max}$ and ${fitness}_{min}$ are the worst and best fitness values in the current generation, respectively; fitness (i) is the current fitness value of the $i^{th}$ search agent. The survival vector in Equation (4) contains normalized fitness values within the interval [0, 1]. For agents with low survival rates (e.g., survival rate ≤ 0.3), the position is updated according to Equation (5):

(5) $$X_{i}\left(t\right)=X_{gbest}+\frac{1}{2}\left[X_{r_{1}}(t)-(-1{)}^{\sigma }\cdot X_{r_{2}}(t)\right]$$

where $X_{i}\left(t\right)$ is the search agent with a low survival rate that requires updating; $X_{r_{1}}(t)$ and $X_{r_{2}}(t)$ are the selected ${r_{1}}^{th}$ and ${r_{2}}^{th}$ search agents, respectively; $X_{gbest}$ is the best search agent found in the previous iteration; σ is a randomly generated binary number.

### 2.2. Hybrid Strategy Improved Dingo Optimization Algorithm (HSIDOA)

To address the limitations of the standard Dingo Optimization Algorithm (DOA)—such as susceptibility to local optima in later iterations, insufficient convergence accuracy, and inefficient handling of out-of-bounds solutions. This study proposes a Hybrid Strategy Improved Dingo Optimization Algorithm (HSIDOA). HSIDOA integrates a quadratic interpolation search strategy, a horizontal crossover search strategy, and a centroid-based opposition learning boundary-handling strategy, enhancing the algorithm from three dimensions: local exploitation, global exploration, and boundary constraint management. This framework aims to achieve a balance between search accuracy and convergence speed.

#### 2.2.1. Quadratic Interpolation Search Strategy

In the standard DOA, the scavenging behavior generates new solutions solely based on the linear combination of random individuals, lacking the ability to exploit nonlinear relationships among individuals. This limitation weakens local exploitation, particularly in complex high-dimensional problems, often causing the algorithm to miss the neighborhood of optimal solutions. To address this, HSIDOA introduces a quadratic interpolation search strategy, which constructs a quadratic interpolation model based on the global best solution and randomly selected high-quality solutions. This allows precise targeting of local optimum regions and strengthens the algorithm’s local exploitation capability.

The quadratic interpolation search strategy focuses on uncovering the nonlinear guidance value of high-quality solutions in the population. The implementation logic is as follows [32]: During the scavenging phase (when a random probability ≥ 0.5), two dingo individuals different from the current agent (denoted as $X_{p}$ and $X_{q}$) are randomly selected. Simultaneously, the global best solution $X_{gbest}$ and the current individual $X_{i}$ are extracted. The corresponding fitness values $f_{p},\;f_{q}\;and\;f_{\mathrm{gbest}}$ are then obtained to construct a quadratic interpolation function, fitting the distribution characteristics of high-quality solutions. Finally, the optimal interpolation point is calculated using the interpolation formula, which serves as the new solution for the scavenging behavior, enabling precise exploration of high-quality local regions.

The core computation of the quadratic interpolation is expressed as [32]:

(6) $$X_{\mathrm{interp}\;}(j)=0.5\cdot \frac{(X_{p}(j{)}^{2}-X_{q}(j{)}^{2})\cdot f_{\mathrm{gbest}\;}+(X_{q}(j{)}^{2}-X_{i\;}(j{)}^{2})\cdot f_{p}+\left(X_{i\;}(j{)}^{2}-X_{p}(j{)}^{2}\right)\cdot f_{q}}{(X_{p}(j)-X_{q}(j))\cdot f_{\mathrm{gbest}\;}+\left(X_{q}(j)-X_{i\;}(j)\right)\cdot f_{p}+\left(X_{i\;}(j)-X_{p}(j)\right)\cdot f_{q}}$$

where j is the dimension index (j=1,2,…,dim), and $X_{\mathrm{interp}\;}$ denotes the interpolation result in the $j^{th}$ dimension.

To prevent numerical singularities when the denominator approaches zero, a singular value handling mechanism is introduced:

(7) Xi,jt+1=Xinterp j, if  denominatorj≥εXgbestj, if  denominatorj<εdenominatorj=(Xp(j)−Xq(j))·fgbest +Xq(j)−Xi (j)·fp+Xi (j)−Xp(j)·fq

Here, ε is a small constant, and $X_{gbest}$ is the $j^{th}$ dimension of the global best solution, ensuring that singular dimensions still move toward high-quality regions.

#### 2.2.2. Horizontal Crossover Search Strategy

In the standard DOA, population updates rely solely on individual-level hunting or scavenging behaviors, lacking a population-level information exchange mechanism. This results in a rapid loss of diversity and a tendency to fall into local optima in later iterations. To address this, HSIDOA introduces a horizontal crossover search strategy, which facilitates complementary information exchange through nonlinear crossover between pairs of individuals, enhancing global exploration while preserving high-quality solutions.

The horizontal crossover search strategy focuses on improving the population’s global exploration capability and information-sharing efficiency. The implementation logic is as follows [33]: After individual updates in each iteration, the population indices are randomly shuffled, and individuals are paired as parents (avoiding fixed pairings that may limit search scope). Then, a nonlinear crossover formula is applied to each parent pair to generate offspring, introducing random weights and shift coefficients to balance exploration and exploitation. Finally, only offspring with fitness superior to their parents are retained, preventing inferior solutions from entering the population, while the global best solution is updated to ensure high-quality solutions are preserved [33].

(8) Xchild1=λ1·Xparent1+1−λ1·Xparent2+μ1·Xparent1−Xparent2Xchild2=λ2·Xparent2+1−λ2·Xparent1+μ2·Xparent2−Xparent1

where $\lambda_{1},\;\lambda_{2}\sim U(0,\;1)$ are dimension-level random weights; $\mu_{1},\;\mu_{2}\sim U(-1,\;1)$ are dimension-level shift coefficients; $X_{parent1}$ and $X_{parent2}$ are the parent individuals, while $X_{child1}$ and $X_{child2}$ are the offspring individuals. This formula nonlinearly combines parental information, preserving high-quality traits from the parents while introducing random perturbations to enhance population diversity.

#### 2.2.3. Centroid-Based Opposition Learning Boundary-Handling Strategy

In the standard DOA, out-of-bounds solutions are handled using a simple boundary truncation approach (directly resetting values to the boundary), which completely discards the information contained in the out-of-bounds solutions. This often causes the population to converge toward the boundaries, reducing search efficiency. To address this, HSIDOA introduces a centroid-based opposition learning boundary-handling strategy, which reconstructs out-of-bounds solutions through centroid reflection. This approach preserves the directional information of the solutions while ensuring their feasibility, thereby enhancing the search capability in boundary regions.

The centroid-based opposition learning boundary-handling strategy focuses on efficiently utilizing the potential information of out-of-bounds solutions. The implementation logic is as follows: First, compute the centroid of the current population (i.e., the mean of all individuals across each dimension), serving as the global reference point of the population. Next, identify all out-of-bounds dimension values (values below the lower bound $lb\left(j\right)$ or above the upper bound $ub\left(j\right)$, and reconstruct these dimensions using the centroid-based reflection formula. Finally, apply a secondary boundary truncation to ensure that all reconstructed solutions fall within the feasible range.

The core computation of centroid-based reflection is given by:

(9) $$X\left(i,j\right)=\left\{\begin{array}{cc} 2\cdot X_{centroid}\left(j\right)-X_{i}\left(j\right), & if\;X\left(i,j\right)<lb\left(j\right)\;or\;X\left(i,j\right)>ub\left(j\right)\\ X\left(i,j\right), & otherwise \end{array}\right.$$

where $X_{centroid}\left(j\right)=\frac{1}{Pop}\sum_{k=1}^{Pop}X\left(k,j\right)$ is the centroid of the $j^{th}$ dimension of the population (Pop denotes the population size); $X\left(i,j\right)$ is the $j^{th}$ dimension of the $i^{th}$ individual; $lb\left(j\right)$ and $ub\left(j\right)$ are the lower and upper bounds of the $j^{th}$ dimension.

This strategy pulls out-of-bounds solutions back into the feasible region while preserving their directional features relative to the population centroid, avoiding information loss caused by simple truncation and enhancing the algorithm’s ability to explore high-quality solutions near boundaries.

In summary, the pseudocode of the proposed HSIDOA is presented in Algorithm 1.
***Algorithm 1:*** *The pseudo-code of the HSIDOA**1: Initialization of parameters.* *2:* 
P=0.5*, probability of hunting or scavenger strategy.* *3:* 
Q=0.7*, probability of Strategy1 (groupattack) or Strategy2 (persecution attack).* *4: Initialization population* 
X. *5: **while*** 
t <T ***do*** *6:*   
$\beta_{1}=-2+2\cdot rand,\;\beta_{2}=-1+2\cdot rand$ *7:   **for*** 
i=1:N ***do*** *8:     **if*** 
rand<P ***do*** *9:      **if*** 
rand<Q ***do*** *10:       Strategy1: Update* 
$X_{i,j}\left(t+1\right)$
*by Equation (1).* *11:      **else*** *12:       Strategy2: Update*
$X_{i,j}\left(t+1\right)$
*by Equation (2).* *13:      **end if*** *14:     **else*** *15:      Strategy3: Update* 
$X_{i,j}\left(t+1\right)$ *by Equation (6) and Equation (7) (Quadratic interpolation).* *16:     **end if*** *17:     % Survival mechanism* *18:     **if*** 
$survival\left(i\right)<\;0.3$
***do*** *19:      Strategy2: Update* 
$X_{i,j}\left(t\right)$ *by Equation (5)*. *20:     **end if*** *21:    Update* 
$X_{i,j}$ *by Equation (8) (Horizontal crossover strategy).* *22:     Perform boundary checking and handling using Equation (9)*. *23:    **end for*** *24:   * 
t=t+1 *25: **end while*** *26: **return** the best solution* 
$X_{gbest}$.

It should be noted that HSIDOA is a stochastic optimization algorithm. Random numbers are generated in several stages of the algorithm, including:

(1) The random selection of search agents in the group attack and pursuit strategies (Equations (1)–(3));
(2) The generation of random coefficients and binary variables controlling movement directions and survival decisions (Equations (2), (3) and (5));
(3) The random selection of individuals and weights in the quadratic interpolation strategy (Equations (6) and (7)); and
(4) The random weights and shift coefficients used in the horizontal crossover strategy (Equation (8)).

These stochastic components ensure population diversity and enhance global exploration capability.

### 2.3. Complexity Analysis of HSIDOA

In the complexity analysis of HSIDOA, let N denote the population size, D the dimension of the optimization problem, and T the maximum number of iterations. The initialization stage of HSIDOA, including population generation and initial fitness calculation, has a time complexity of O(N×D), which is identical to that of the standard DOA and does not introduce additional computational overhead. During each iteration, HSIDOA retains the core behavioral logic of DOA (hunting, survival mechanism) with a complexity of O(N×D), and integrates three enhanced strategies: the quadratic interpolation search strategy involves vectorized calculation of interpolation parameters for each individual, with a per-iteration complexity of O(N×D); the horizontal crossover strategy implements pairwise nonlinear crossover for the population, with a complexity of O(N×D) after vectorization optimization; the centroid reflection boundary control strategy completes boundary adjustment through vectorized judgement and reflection calculation, with a per-iteration complexity of O(N×D). Although multiple enhanced strategies are added, all strategies adopt vectorized operations to avoid nested loops, so the total complexity of each iteration of HSIDOA remains O(N×D). Combined with T iterations, the overall time complexity of HSIDOA is O(T×N×D), which is consistent with the standard DOA. This means HSIDOA achieves the improvement of optimization precision and convergence speed without increasing the algorithm’s computational complexity, ensuring its applicability in high-dimensional optimization scenarios.

## 3. Numerical Experiments of CEC2017 and CEC2022

### 3.1. Comparative Methods and Parameter Settings

In this subsection, the proposed HSIDOA algorithm is evaluated using the currently most challenging numerical optimization benchmark, CEC2017 [34] and CEC2022 [35]. Its efficacy is contrasted against multiple sophisticated optimizer techniques. Evaluated competitors comprise: Multi-level Particle Swarm Optimization (MLPSO) [22], Memory, Evolutionary operator, and Local search based improved Grey Wolf Optimizer (MELGWO) [23], Multi-Strategy Hybrid Whale Optimization Algorithm (MHWOA) [24], Artificial Lemming Algorithm (ALA) [36], Hippopotamus Optimization (HO) [37], Rime optimization algorithm (RIME) [38], and standard Dingo Optimization Algorithm (DOA) [28]. Corresponding algorithmic configurations appear consolidated within Table 1.

### 3.2. Ablation Study Assessment

**Figure 2:**
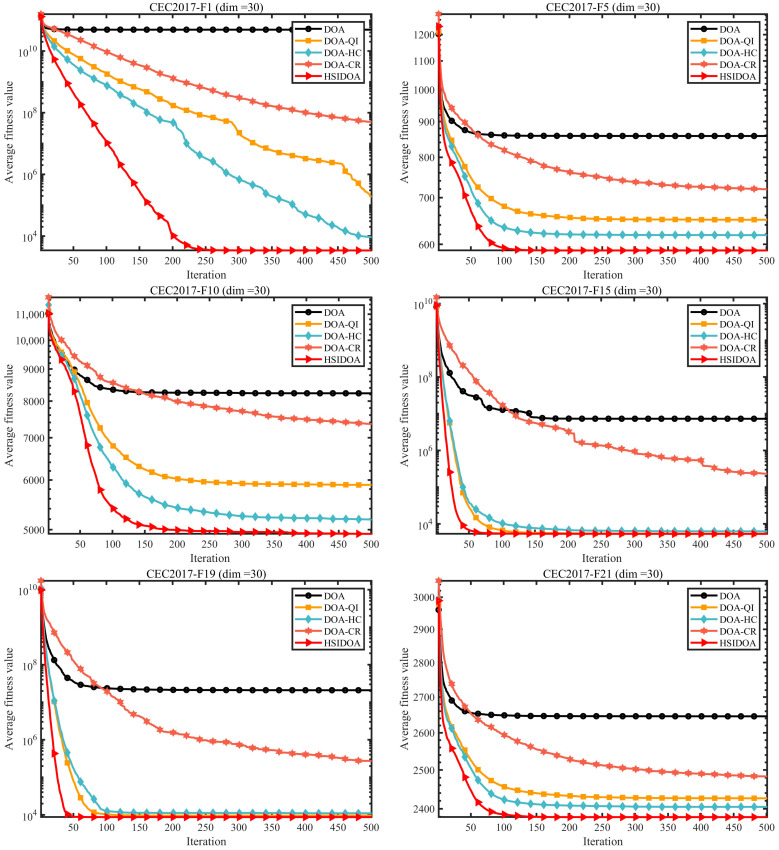

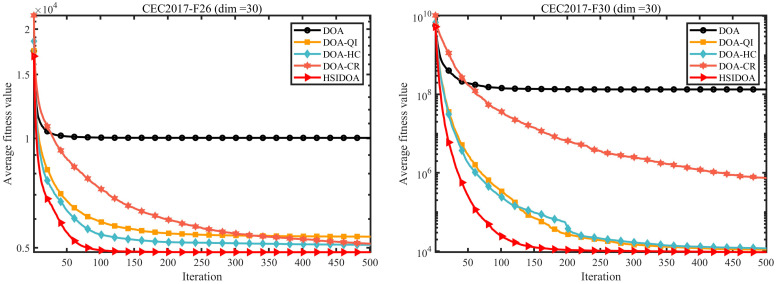
Enhanced DOA versions convergence across different modification approaches.

**Figure 3:**
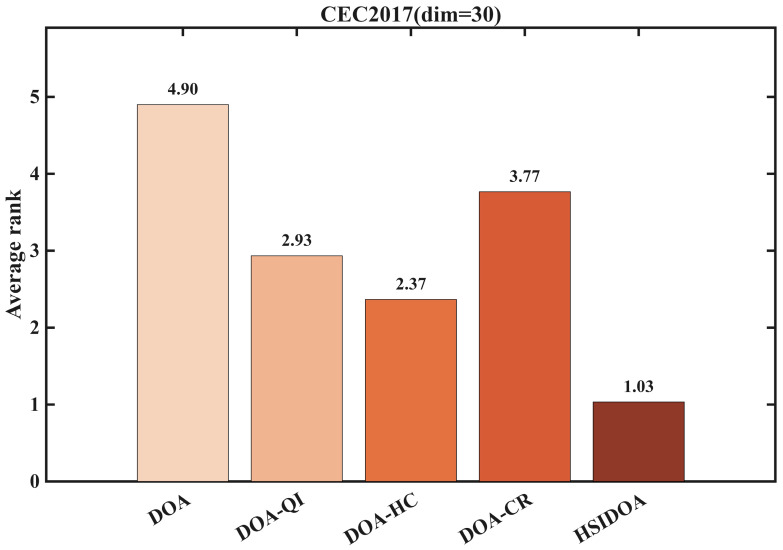
Average order of upgraded DOA models across distinct enhancement techniques.

The performance evaluation was conducted using an ablation experimental design to quantitatively analyze the contribution of each improvement strategy to the DOA algorithm. Experiments were performed on the 30-dimensional CEC2017 benchmark function set. The control group consisted of the original DOA algorithm, while the experimental groups included three variants with individual strategies applied sequentially: DOA-QI (Quadratic Interpolation Search Strategy), DOA-HC (Horizontal Crossover Search Strategy), and DOA-CR (Centroid-Based Opposition Learning Boundary-Handling Strategy), as well as the HSIDOA algorithm incorporating all strategies. Detailed performance comparison data are provided in Table 2, and the trend visualization results are presented in Figure 2 and Figure 3.

The quantitative results in Table 2 indicate that HSIDOA, incorporating all three improvement strategies, significantly outperforms the original DOA and its single-strategy variants (DOA-QI, DOA-HC, and DOA-CR) on most CEC2017 (30D) test functions. Taking representative functions as examples, the mean of F1 decreases from 4.9148E+10 (DOA) to 3.4022E+03 (HSIDOA); F3 drops from 6.9500E+04 to 3.5588E+03; and F12 reduces from 6.8128E+09 to 3.3247E+05. These results demonstrate that HSIDOA can substantially lower the objective values across multiple typical test functions, achieving performance improvements of several orders of magnitude.

In terms of standard deviation, HSIDOA also exhibits markedly higher stability. For instance, the standard deviation of F1 decreases from 9.1464E+09 (DOA) to 4.3862E+03, and that of F3 declines from 9.2972E+03 to 2.7549E+03. The simultaneous improvement in both mean and variance indicates that the integration of the three strategies not only enhances solution accuracy but also strengthens the algorithm’s stability across multiple independent runs, endowing HSIDOA with greater robustness in complex optimization tasks.

The convergence curves in Figure 2 visually illustrate the iterative optimization process of each algorithm. For representative functions such as F1, F5, and F10, the convergence curve of HSIDOA consistently remains at the bottom, indicating superior performance. Moreover, HSIDOA demonstrates faster early-stage convergence, quickly approaching the optimal solution within the initial iterations, whereas the original DOA curve stays higher, converging slowly and showing signs of stagnation. For example, on F10, the HSIDOA fitness value decreases to approximately 5.0E+03 after 50 iterations, while DOA remains above 8.0E+03. After 500 iterations, HSIDOA achieves a fitness improvement of about 40% compared to DOA. Among the three single-strategy variants, DOA-HC and DOA-QI outperform DOA-CR, particularly in the mid-to-late iterations. DOA-HC maintains search momentum through population-level information exchange, while DOA-QI accelerates convergence by precisely exploiting local optima. HSIDOA effectively integrates these advantages, achieving a balance between convergence speed and accuracy.

The average ranking results in Figure 3 further validate the effectiveness of the improvement strategies. HSIDOA attains the best overall performance with an average rank of 1.03, significantly outperforming the original DOA, which ranks 4.90, across the 30 benchmark functions. DOA-HC (2.37) and DOA-QI (2.93) show similar average rankings, both surpassing DOA-CR (3.77), indicating that the horizontal crossover search strategy enhances population diversity and the quadratic interpolation search strategy strengthens local exploitation, which are the main contributors to performance improvement. Although DOA-CR ranks relatively lower, it still outperforms the original DOA, demonstrating that the centroid-based opposition learning boundary-handling strategy effectively preserves out-of-bounds solution information and improves boundary search efficiency, thus providing complementary performance gains.

By comparing DOA-QI, DOA-HC, DOA-CR, and HSIDOA, it can be observed that quadratic interpolation (DOA-QI) and horizontal crossover (DOA-HC) provide significant benefits across most test functions, whereas centroid-based opposition learning (DOA-CR) achieves relatively modest improvements in certain functions. Overall, DOA-QI enhances local exploitation, DOA-HC maintains population diversity, and DOA-CR strengthens boundary handling. When these strategies are synergistically combined, HSIDOA achieves an optimal balance between global exploration and local exploitation, resulting in the best overall performance across the majority of benchmark functions.

### 3.3. Performance Assessment Using CEC2017 and CEC2022 Benchmarks

**Figure 4:**
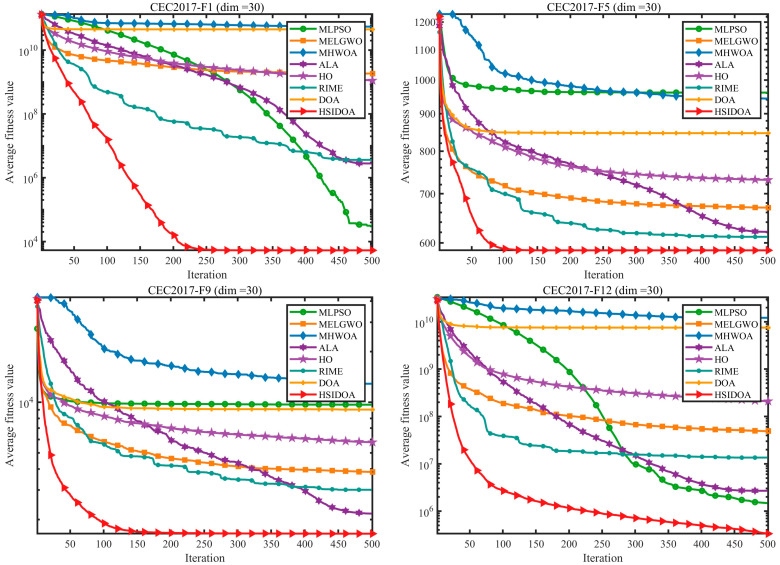

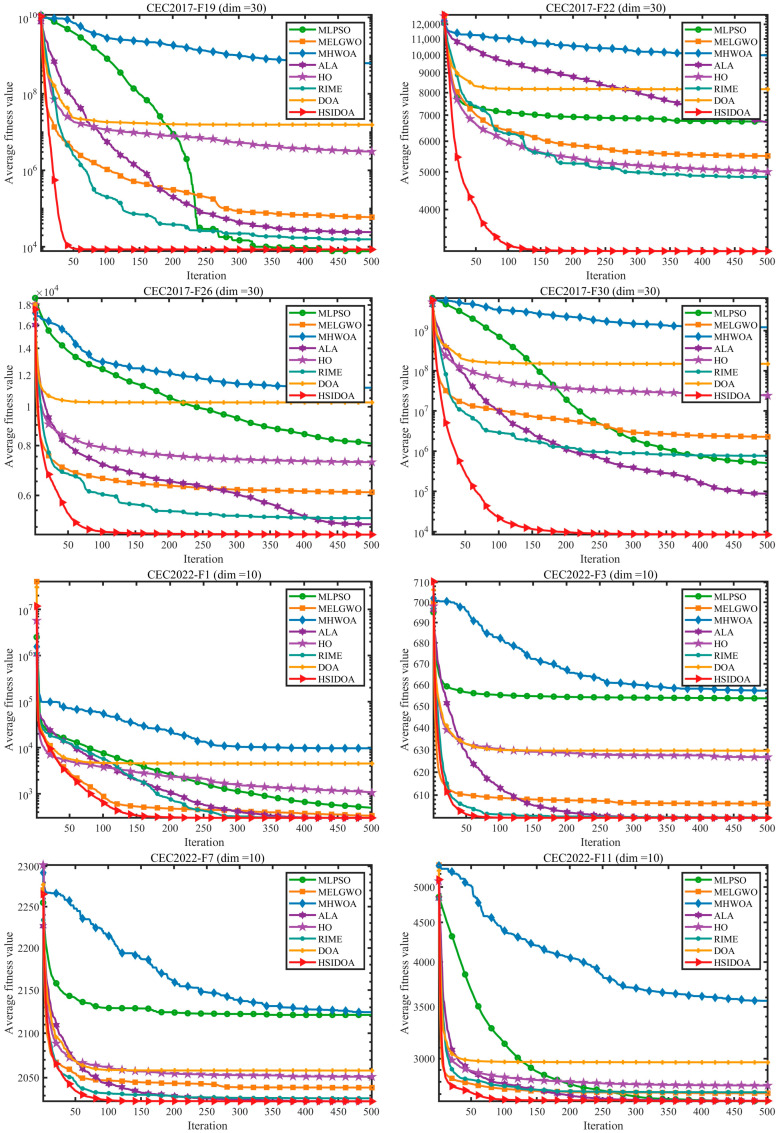

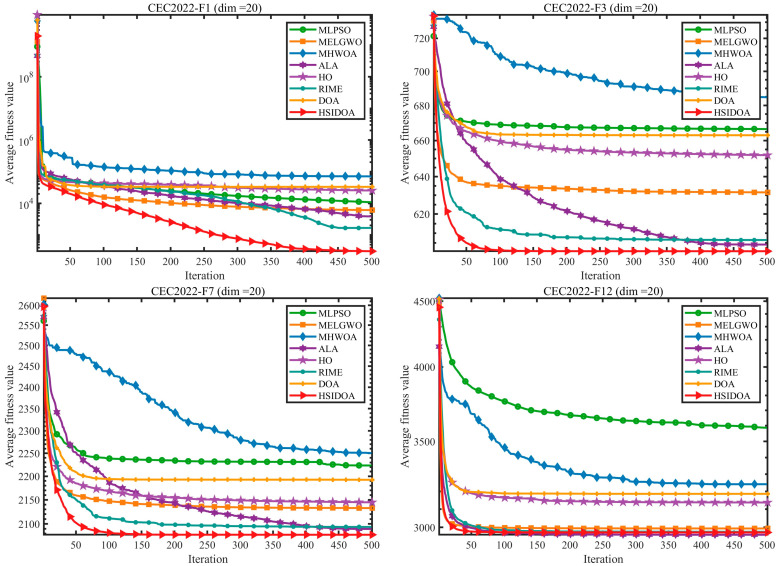
Convergence speed comparison among the tested methods using the benchmark suite.

To further verify the generality and superiority of the HSIDOA algorithm, comprehensive comparisons were conducted on three benchmark function sets: CEC2017 (30-dimensional) and CEC2022 (10-dimensional and 20-dimensional). HSIDOA was compared against seven mainstream optimization algorithms, including MLPSO and MELGWO. The experimental results are presented in Table 3, Table 4 and Table 5, and the convergence visualization is shown in Figure 4. All algorithms were evaluated under a unified experimental setup: a population size of 30, a maximum of 500 iterations, and 30 independent runs for each algorithm, ensuring fairness and reliability of the comparisons. All simulations execute within an identical computational environment: Windows 10 OS, an Intel Core i5-13400 (13th Gen) processor running at 2.5 GHz, 16 GB of system memory, employing MATLAB version 2024b. This consistent setup and evaluation methodology ensures outcome reliability and enables straightforward cross-method examination. Outcome data are presented across Table 3, Table 4, Table 5 and Table 6 and illustrated in Figure 4.

As shown in Table 3, HSIDOA demonstrates a remarkable advantage in 30-dimensional complex optimization problems. For the unimodal function F1, HSIDOA achieves an average fitness value of 5.2966E+03, which is significantly lower than DOA (4.5105E+10), MHWOA (5.4819E+10), and other comparative algorithms, and even outperforms well-performing ALA (2.8014E+06) and RIME (3.6284E+06), representing an improvement in optimization precision of over 500 times. For the multimodal function F9, HSIDOA attains an average fitness of 1.6487E+03, only 18.2% of DOA’s value (9.0304E+03), and lower than MELGWO (3.8466E+03) and RIME (3.0110E+03), illustrating its strong capability to locate global optima in complex multimodal landscapes. In addition, HSIDOA achieves the smallest standard deviations on most functions, such as F6 (Std = 4.3639E+00), outperforming all other algorithms, indicating stable optimization performance even in high-dimensional scenarios.

Table 4 (10 dim) and Table 5 (20 dim) further show that HSIDOA performs outstandingly in low- to medium-dimensional optimization problems. On the 10-dimensional F1 function, HSIDOA reaches an average fitness value of 3.0000E+02, achieving the best performance alongside ALA and RIME, with an extremely low standard deviation of 5.9585E−07, far below other algorithms, demonstrating precise convergence. For the 20-dimensional F5 function, HSIDOA attains an average fitness of 9.8782E+02, significantly outperforming DOA (2.7412E+03), MLPSO (3.9613E+03), and MELGWO (1.6082E+03), and is the only algorithm with an average fitness below 1.0E+03. Notably, as the dimensionality increases from 10 dim to 20 dim, most algorithms exhibit substantial performance degradation (e.g., MHWOA’s F6 average fitness rises from 2.2239E+06 to 2.0072E+09), whereas HSIDOA shows the minimal decline, highlighting its strong dimensional adaptability.

The convergence curves in Figure 4 intuitively illustrate the dynamic optimization characteristics of each algorithm. For functions such as CEC2017-F5 and F9, the HSIDOA consistently maintains the lowest convergence curves and exhibits the fastest convergence speed: during the early iterations (first 50), the fitness values rapidly decrease and approach the optimal solutions, while in the middle iterations (100–200), the curves stabilize, avoiding the stagnation commonly observed in other algorithms during later stages. For example, in the CEC2017-F12 function, the HSIDOA achieves stable convergence by iteration 200, whereas the DOA, MHWOA, and other algorithms continue to decrease slowly, ultimately converging to values significantly higher than those of the HSIDOA.

The convergence advantage of the HSIDOA is also evident in the 10D and 20D functions of the CEC2022 benchmark. In the 10-dimensional F3 function, its convergence curve closely tracks the optimal value with near-zero standard deviation. In the 20-dimensional F7 function, the HSIDOA maintains a convergence speed comparable to the 10D scenario, while algorithms such as MLPSO and DOA exhibit noticeable upward shifts in their convergence curves, demonstrating the HSIDOA’s stable convergence across different dimensionalities. Compared with all other algorithms, the HSIDOA achieves dual optimization in convergence speed and accuracy through local precise search via quadratic interpolation and global information exchange via horizontal crossover, exhibiting superior dynamic optimization performance across unimodal, multimodal, and high-dimensional functions.

In summary, the quantitative results in Table 3, Table 4 and Table 5 and the dynamic convergence behaviors in Figure 3 collectively indicate that the HSIDOA consistently outperforms other algorithms across benchmarks of varying dimensionality, category, and complexity. The synergistic integration of multiple strategies effectively enhances the algorithm’s global exploration capability, local exploitation precision, and intelligent boundary handling. Consequently, the HSIDOA demonstrates significant advantages in convergence speed, search stability, and high-dimensional scalability, confirming its potential as a powerful optimization framework for tackling complex real-world problems.

### 3.4. Runtime Comparison Between the DOA and HSIDOA

**Figure 5:**
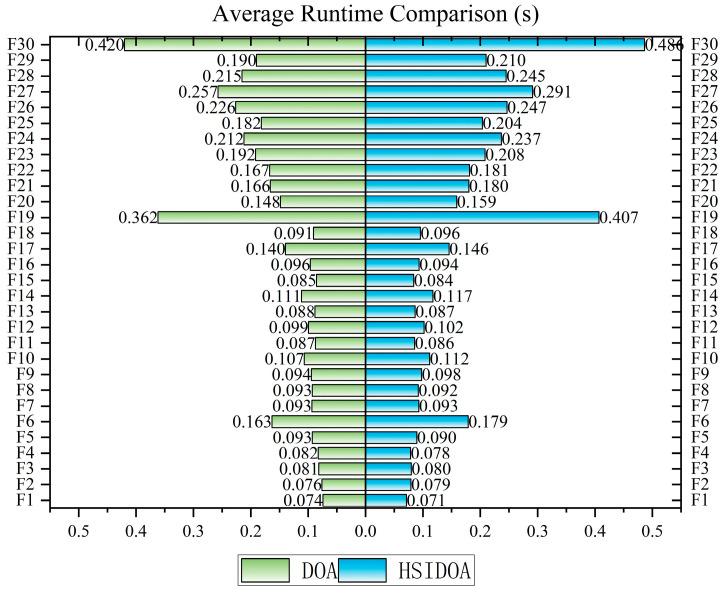
Comparison of average runtime between the DOA and HSIDOA on CEC2017 (dim = 30).

To accurately evaluate the computational efficiency of the HSIDOA after incorporating the three enhancement strategies, this section conducts a quantitative comparison of the average runtime between the HSIDOA and the original DOA using the CEC2017 benchmark function set (30 dimensions). The experimental data are summarized in the corresponding table. All tests were performed under the same hardware (Intel Core i5-13400 processor, 16 GB RAM) and software (MATLAB 2024b) environment to ensure fairness and reliability of the comparison. The experimental results are illustrated in Figure 5.

From the experimental results, it can be observed that the overall runtime of the HSIDOA is on the same order of magnitude as that of the DOA. Only for the F1 function does the HSIDOA exhibit a slightly shorter runtime (0.0714 s) than the DOA (0.0740 s). For the remaining 29 functions, the runtime of the HSIDOA increases slightly, but the increments are kept within a reasonable range. For most functions, the runtime increase does not exceed 10%. For example, on the F3 function, the runtime of the HSIDOA is 0.0799 s, representing only a 1.8% increase compared with the DOA’s 0.0814 s; on the F5 function, the HSIDOA runs in 0.0896 s, which is 3.4% higher than the DOA’s 0.0928 s—well within the tolerance of computational overhead in engineering applications. Even for complex multimodal functions (e.g., F19 and F30), the runtime increase of the HSIDOA does not exceed 16%. Specifically, for F19, the HSIDOA requires 0.4071 s, a 12.5% increase over DOA’s 0.3618 s; for F30, the HSIDOA runs in 0.4864 s, which is 15.8% higher than DOA’s 0.4201 s.

It is worth noting that the slight increase in the HSIDOA’s runtime is a reasonable cost for introducing three strategies: quadratic interpolation search, horizontal crossover search, and centroid opposition-based learning boundary handling. In terms of performance gains, the HSIDOA achieves order-of-magnitude improvements in optimization accuracy, convergence speed, and stability. For instance, the mean fitness value on the F1 function decreases from 4.9148E+10 with DOA to 3.4022E+03 with the HSIDOA, and on the F3 function from 6.9500E+04 to 3.5588E+03. This trade-off—accepting a slight increase in computational time in exchange for significant performance improvements—has substantial engineering value.

From a complexity perspective, all enhancement strategies in the HSIDOA are implemented using vectorized operations, thereby avoiding the efficiency loss caused by nested loops. As a result, the overall time complexity remains *O(T × N × D)*, which is identical to that of the original DOA. The minor differences in runtime mainly stem from additional vector computations and logical checks during strategy execution rather than from an increase in theoretical complexity. These results demonstrate that the HSIDOA achieves comprehensive performance optimization through well-designed strategies without increasing algorithmic complexity, effectively balancing computational efficiency and optimization performance and exhibiting strong practical applicability in engineering contexts.

### 3.5. Statistical Evaluation via Friedman Test

To comprehensively evaluate the HSIDOA method’s relative standing, a statistical measure suitable for simultaneously comparing several interrelated algorithms is required. For this objective, the Friedman test—a nonparametric technique—analyzes different optimizers according to their ranked performance over multiple problem sets. This methodology intrinsically avoids presuppositions about the results’ probability distribution, making it especially effective for assessing multiple techniques against the same suite of benchmark tasks. The corresponding test statistic is derived from the formula provided in the references [8,39]:

(10) $$Q=\frac{12}{kn\left(k+1\right)}\sum_{j=1}^{k}R_{j}^{2}-3n\left(k+1\right)$$

In the given equation, n represents block count, k indicates group quantity, and $R_{j}$ corresponds to the aggregate ordinal position for the $j^{th}$ group. Provided both n and k fulfill requisite size criteria, the resulting Q value follows a chi-square distribution characterized by k−1 degrees of liberty [39].

**Figure 6:**
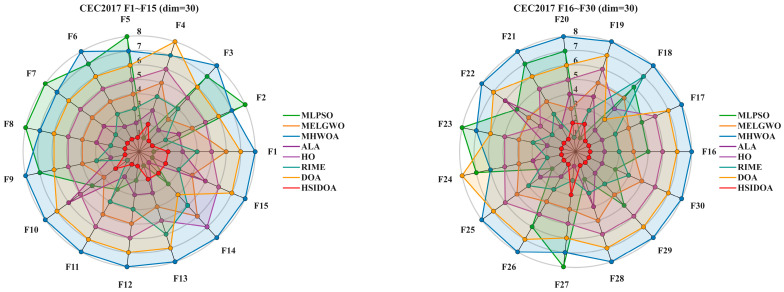

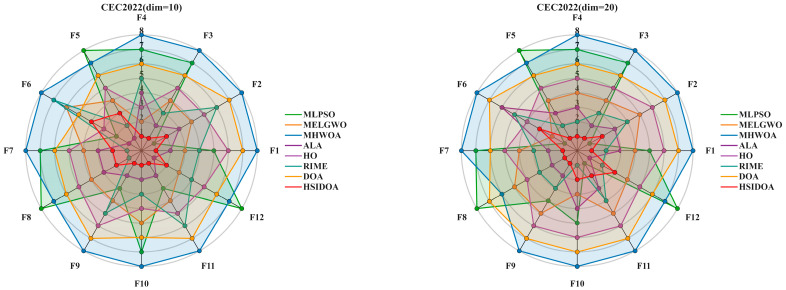
Graphical representation of relative performance distribution across the evaluated algorithm collection.

To statistically validate the superior performance of the HSIDOA, the Friedman test was conducted to analyze the significance of ranking results across all compared algorithms on the CEC2017 (30D) and CEC2022 (10D and 20D) benchmark function sets. The detailed ranking data are presented in Table 6, and the distribution of rankings is visualized in Figure 6.

From the summary of Friedman rankings (Table 6), it is evident that the proposed the HSIDOA achieves the best average ranks across all three experimental setups (CEC2017 30D, CEC2022 10D, and 20D), with values of 1.33, 1.75, and 1.58, respectively, consistently securing the first position among all compared algorithms. In contrast, the original DOA ranks at the bottom in all three experiments, with average ranks of 6.47, 6.25, and 6.58, while other advanced algorithms such as MHWOA, MLPSO, and MELGWO occupy intermediate or lower positions. These results indicate that, based on the Friedman ranking, the HSIDOA demonstrates significant overall superiority on these benchmark sets.

Further observation of Table 6 shows that ALA consistently maintains a leading second place (2.80, 2.17, 2.83), suggesting stable competitiveness across these datasets. RIME generally ranks in the upper-middle range (3.20, 3.92, 2.83), indicating advantages under certain experimental configurations. In contrast, MHWOA ranks eighth in all three experiments, reflecting insufficient stability or convergence performance under the benchmark and parameter settings employed in this study.

Figure 6 presents a visualization of the relative ranking distribution of all algorithms, providing an intuitive verification of the tabulated results. The ranking distribution of the HSIDOA is clearly concentrated at the top of the graph (mostly rank 1), indicating not only excellent average performance but also consistent high-ranking outcomes across multiple independent runs. Conversely, algorithms such as the DOA and MHWOA exhibit broader distributions across the middle and lower regions, reflecting both lower average ranks and greater variability.

In summary, the Friedman ranking data in Table 6 and the ranking distribution visualization in Figure 4 jointly demonstrate that the proposed the HSIDOA exhibits significant and stable performance improvements across the selected benchmarks (CEC2017 and CEC2022 with different dimensional settings). In a manuscript, it is recommended to describe the experimental results by reporting both the average ranks to quantify superiority and the ranking distribution to emphasize consistency, thereby providing both quantitative and visual evidence to support the HSIDOA’s advantages.

## 4. Multilevel Thresholding Image Segmentation

To assess the real-world efficacy of the Adaptive Multi-strategy DOA Optimizer (HSIDOA) for hierarchical multilevel image thresholding, comparative trials are structured in this section. Employing Otsu’s inter-class variance maximization criterion as the fitness measure, partitioning experiments are performed on various standard test images across distinct threshold counts. Outcomes are measured and examined through several quantitative performance indices.

Otsu’s method is selected as the objective function in this study because it directly maximizes the between-class variance, which has a clear physical interpretation and strong robustness against noise. Compared with entropy-based methods such as Kapur entropy, Masi entropy, and cross entropy, Otsu’s criterion exhibits lower computational complexity and more stable performance in multi-threshold segmentation tasks [40].

Moreover, Otsu’s method does not require logarithmic operations or probability density estimation, making it particularly suitable for integration with population-based metaheuristic optimization algorithms.

### 4.1. Evaluation Metrics

To effectively evaluate the image segmentation performance of different algorithms, we employ image quality assessment metrics including Peak Signal-to-Noise Ratio (PSNR), Structural Similarity Index (SSIM), and Feature Similarity Index (FSIM). The descriptions of these metrics are presented in Table 7.

### 4.2. Experimental Design

**Figure 7:**
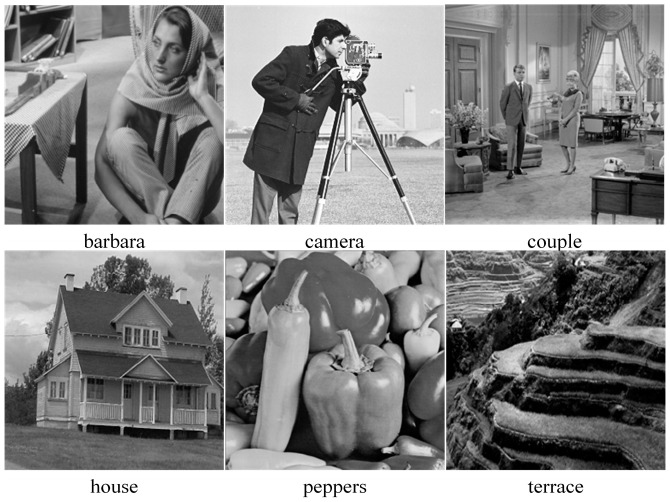
Reference pictures across diverse styles.

To validate the effectiveness of the HSIDOA in practical applications, multi-level thresholding experiments were conducted on six benchmark images with different styles (barbara, camera, couple, etc.), as shown in Figure 7, using Otsu’s maximum between-class variance as the objective function. Four, six, eight, and ten-level thresholding experiments were performed. To ensure a fair comparison, eight mainstream swarm intelligence algorithms, including Particle Swarm Optimization (PSO), Grey Wolf Optimizer (GWO), and the Whale Optimization Algorithm (WOA), were selected as baseline methods. For all algorithms, the population size was set to Pop = 30 and the maximum number of iterations to T = 100, with other parameters consistent with the previous experimental settings.

In this research, Otsu’s inter-class variance maximization technique serves as the fitness criterion for threshold selection. The fundamental concept involves identifying ideal segmentation points through optimizing separation between class variances.

For a digital image containing L distinct intensity levels, the likelihood of a pixel possessing grayscale value i equals:

(11) $$P_{i}=\frac{n_{i}}{N}$$

where $n_{i}$ is the number of pixels with gray level i, and $N=\sum_{i=0}^{L-1}n_{i}$ is the total number of pixels. It satisfies $P_{i}\geq 0,\;and\;P_{0}+P_{1}+\ldots +P_{L-1}=1$.

If k thresholds $T_{1}<T_{2}<\ldots <T_{k}$ are set, the image is divided into k+1 regions. The pixel proportion, mean gray level, overall image mean, and between-class variance of the $i^{th}$ region are defined as [5,43]:

(12) $$\omega_{i}=\sum_{j=T_{i-1}+1}^{T_{i}}P_{j}(T_{0}=-1,T_{k+1}=L-1)$$

(13) $$\mu_{i}=\frac{1}{\omega_{i}}\sum_{j=T_{i-1}+1}^{T_{i}}j\times P_{j}$$

(14) $$\mu =\sum_{i=0}^{k}\omega_{i}\mu_{i}$$

(15) $$\sigma^{2}=\sum_{i=0}^{k}\omega_{i}{\left(\mu_{i}-\mu \right)}^{2}$$

The optimal threshold combination $T_{\mathrm{best}\;}=(T_{1}^{*},T_{2}^{*},\ldots ,T_{k}^{*})$ is the set of thresholds that maximizes the between-class variance:

(16) $$T_{\mathrm{best}\;}=arg\underset{T_{1},T_{2},\ldots ,T_{k}}{max}\sigma^{2}$$

In this study, the inter-class variance refers specifically to the between-class variance defined in Otsu’s method.

### 4.3. Experimental Results and Analysis

**Figure 8:**
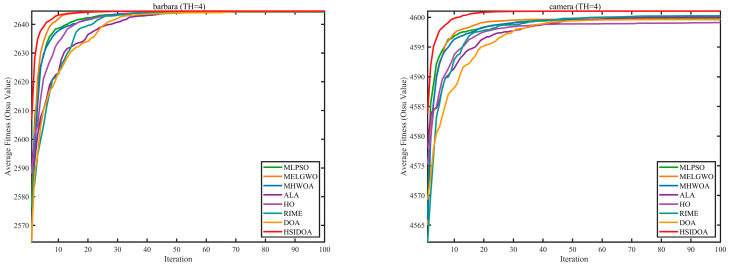

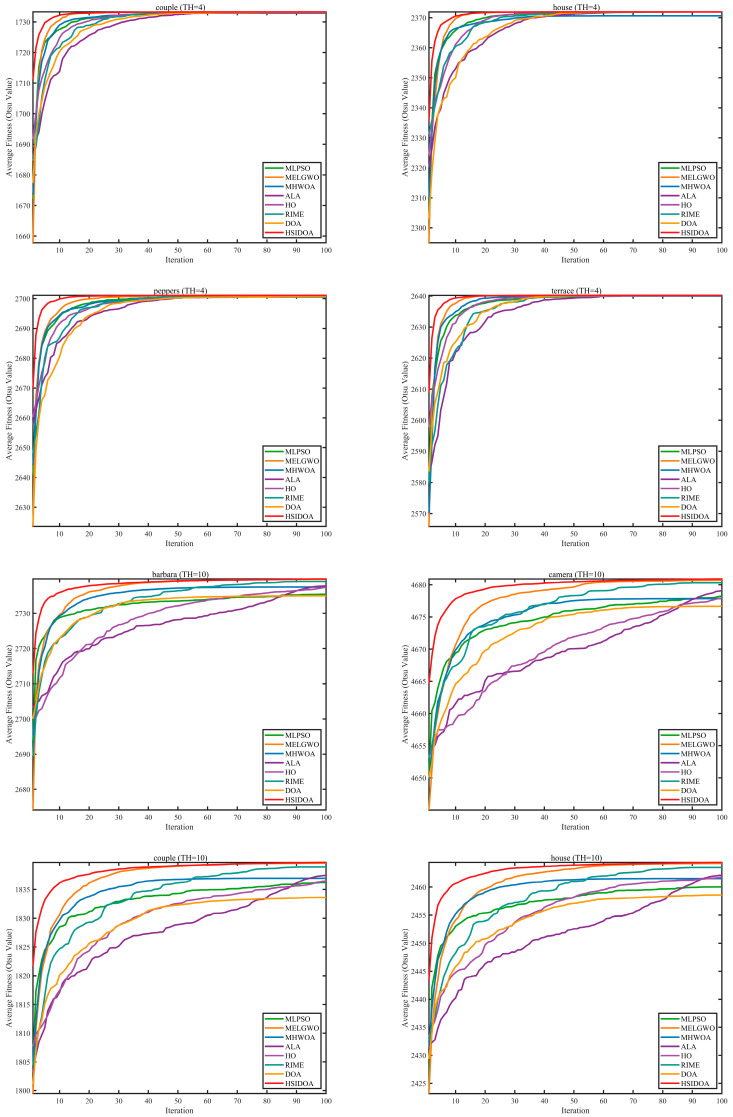

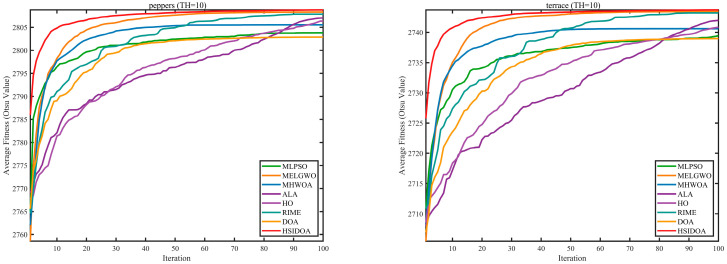
Fitness progression plots for various algorithms on multiple images (TH = 4/10).

**Figure 9:**
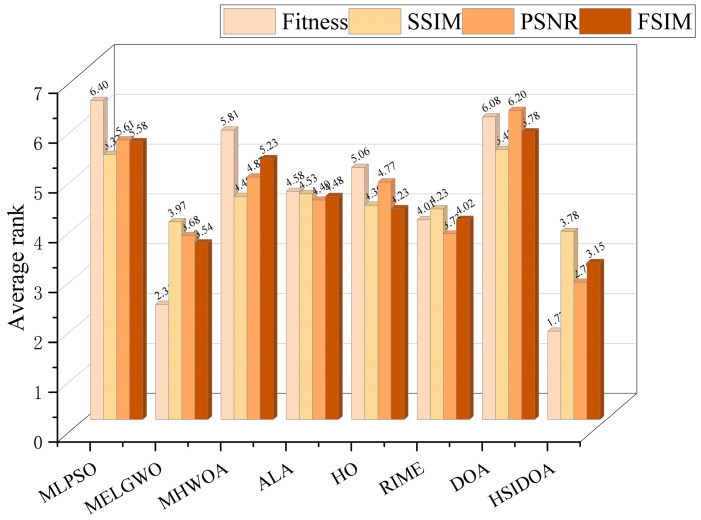
Mean ordinal positions per algorithm under varied evaluation measures.

In this section, a comprehensive comparison is conducted using four key metrics: optimal fitness value, PSNR, SSIM, and FSIM. The HSIDOA is evaluated against seven other algorithms, including MLPSO and MELGWO. The experimental results are summarized in Table 8, Table 9, Table 10, Table 11 and Table 12, while the fitness curves and metric-in Figure 8 and Figure 9.

**Table 8:** Multilevel thresholding results using Otsu’s criterion as the fitness function.

Images	TH = 4	TH = 6	TH = 8	TH = 10
barbara	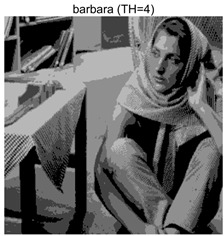	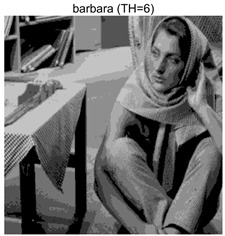	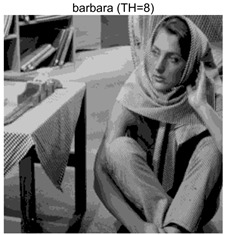	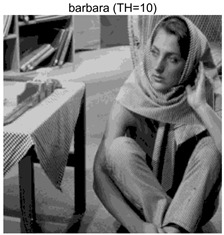
	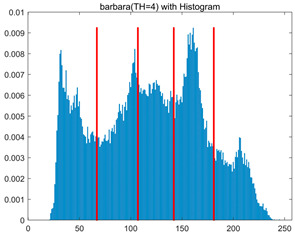	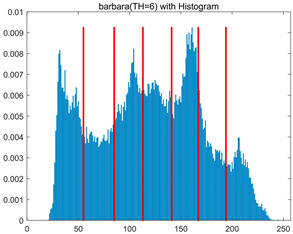	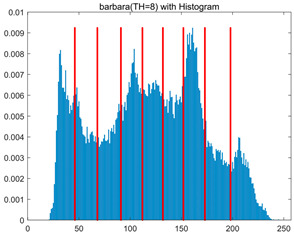	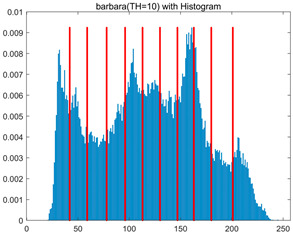
camera	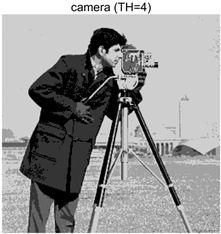	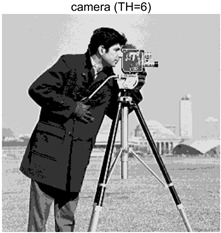	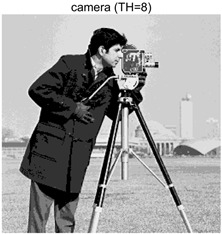	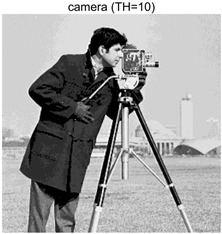
	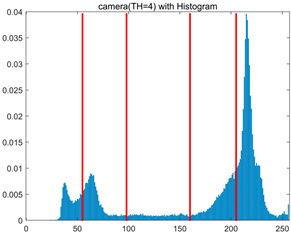	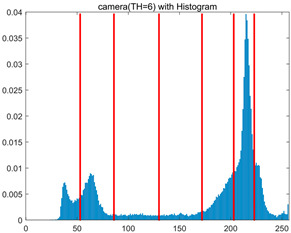	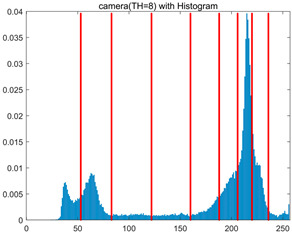	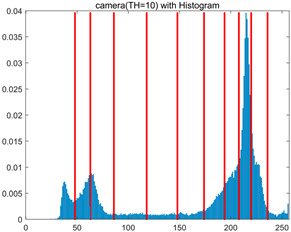
couple	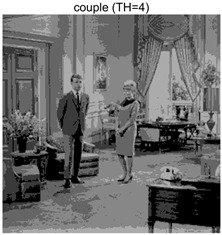	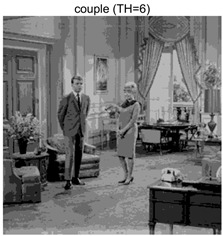	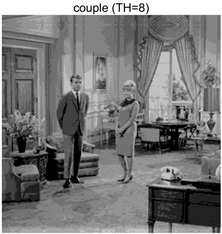	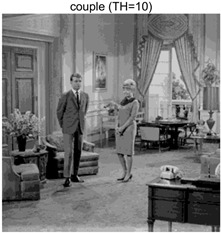
	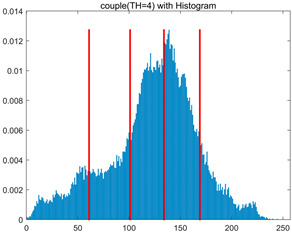	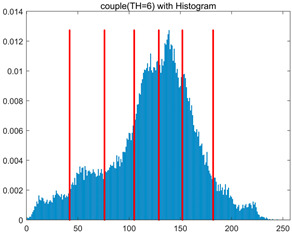	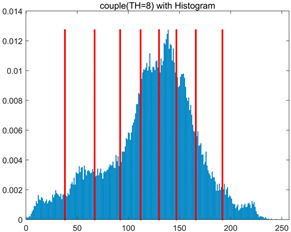	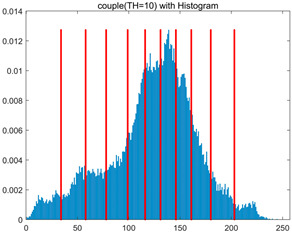
house	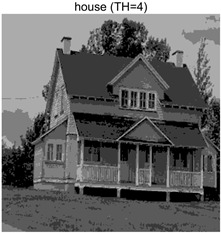	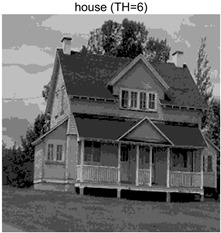	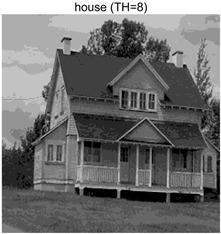	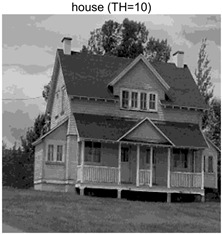
	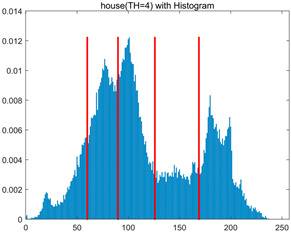	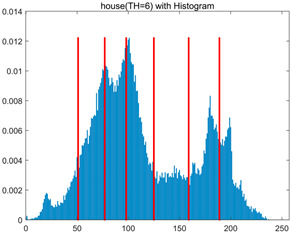	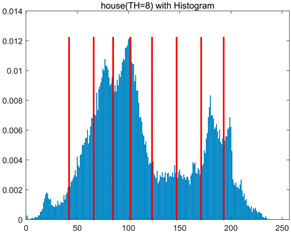	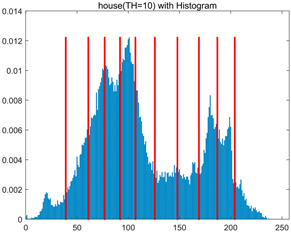
peppers	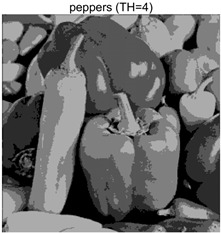	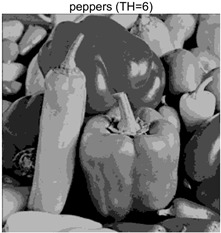	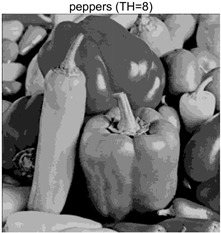	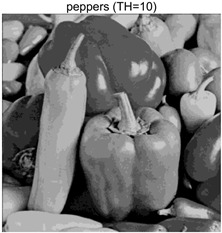
	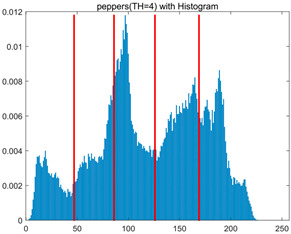	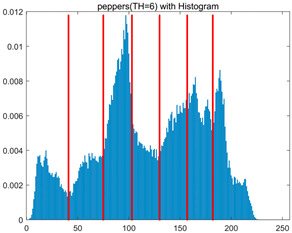	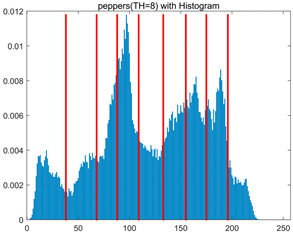	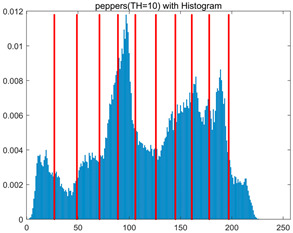
terrace	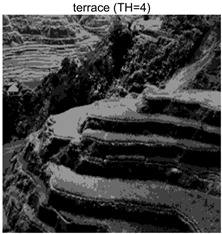	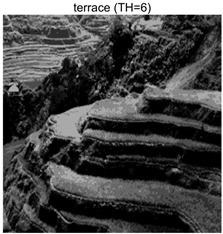	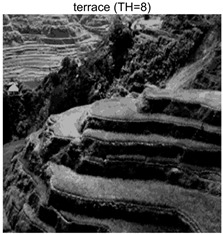	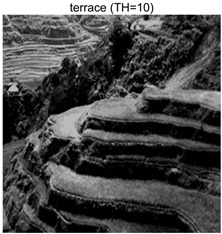
	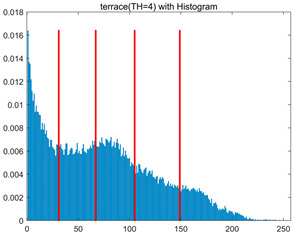	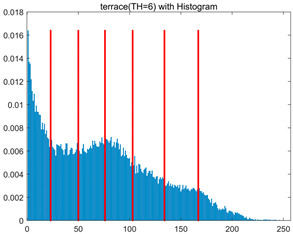	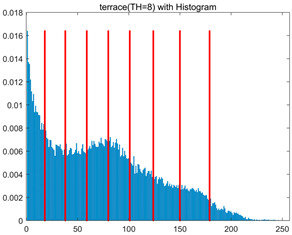	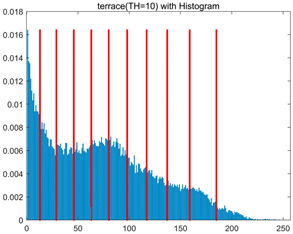

**Table 9:** Mean and Standard Deviation of best Otsu-criterion fitness scores.

Images	TH	Metrics	MLPSO	MELGWO	MHWOA	ALA	HO	RIME	DOA	HSIDOA
barbara	4	Mean	2.6446E+03	2.6446E+03	2.6445E+03	2.6446E+03	2.6446E+03	2.6446E+03	2.6446E+03	2.6446E+03
		Std	1.8501E−12	1.8501E−12	1.1825E−01	1.8501E−12	1.0179E−01	1.8576E−02	1.1312E−02	1.8501E−12
	6	Mean	2.7013E+03	2.7015E+03	2.7013E+03	2.7015E+03	2.7012E+03	2.7015E+03	2.7003E+03	2.7015E+03
		Std	2.2827E−01	4.3652E−02	1.9625E−01	1.1196E−01	5.8174E−01	3.1921E−02	1.7998E+00	6.6734E−03
	8	Mean	2.7246E+03	2.7273E+03	2.7252E+03	2.7262E+03	2.7257E+03	2.7270E+03	2.7231E+03	2.7273E+03
		Std	1.7000E+00	8.2424E−02	2.6157E+00	9.9594E−01	1.8616E+00	2.2665E−01	3.0328E+00	2.2218E−02
	10	Mean	2.7354E+03	2.7397E+03	2.7375E+03	2.7378E+03	2.7373E+03	2.7390E+03	2.7350E+03	2.7398E+03
		Std	1.7997E+00	2.6102E−01	1.5373E+00	1.2587E+00	2.4349E+00	5.3751E−01	2.0969E+00	6.0808E−02
camera	4	Mean	4.5999E+03	4.5997E+03	4.5996E+03	4.6000E+03	4.5991E+03	4.6003E+03	4.5997E+03	4.6011E+03
		Std	1.1002E+00	9.3601E−01	9.9506E−01	1.2217E+00	1.5154E+00	1.1147E+00	1.1793E+00	6.7739E−03
	6	Mean	4.6512E+03	4.6517E+03	4.6499E+03	4.6512E+03	4.6508E+03	4.6515E+03	4.6489E+03	4.6517E+03
		Std	3.2187E−01	1.2312E−02	1.3486E+00	5.3850E−01	2.6366E+00	1.1119E−01	4.2572E+00	9.2504E−13
	8	Mean	4.6681E+03	4.6698E+03	4.6681E+03	4.6686E+03	4.6691E+03	4.6695E+03	4.6663E+03	4.6705E+03
		Std	1.0513E+00	8.7608E−01	1.7729E+00	1.3276E+00	1.0017E+00	8.4293E−01	2.5297E+00	4.0915E−01
	10	Mean	4.6782E+03	4.6807E+03	4.6778E+03	4.6791E+03	4.6779E+03	4.6803E+03	4.6767E+03	4.6809E+03
		Std	1.2825E+00	1.9677E−01	1.8599E+00	1.2302E+00	3.1080E+00	4.2975E−01	2.2110E+00	4.0804E−02
couple	4	Mean	1.7332E+03	1.7333E+03	1.7329E+03	1.7333E+03	1.7331E+03	1.7333E+03	1.7332E+03	1.7333E+03
		Std	5.5630E−02	6.7390E−03	9.8805E−01	7.3788E−02	3.7494E−01	3.6828E−02	1.7161E−01	1.4427E−02
	6	Mean	1.7981E+03	1.7991E+03	1.7970E+03	1.7989E+03	1.7981E+03	1.7990E+03	1.7966E+03	1.7992E+03
		Std	7.5436E−01	9.3963E−03	4.9228E+00	3.5548E−01	1.4095E+00	1.0839E−01	3.0856E+00	1.7133E−03
	8	Mean	1.8238E+03	1.8262E+03	1.8234E+03	1.8255E+03	1.8255E+03	1.8260E+03	1.8232E+03	1.8263E+03
		Std	1.3559E+00	7.7599E−01	4.0845E+00	6.2877E−01	1.0417E+00	3.8584E−01	2.5732E+00	2.8180E−02
	10	Mean	1.8362E+03	1.8396E+03	1.8369E+03	1.8374E+03	1.8364E+03	1.8389E+03	1.8336E+03	1.8397E+03
		Std	1.3290E+00	2.9857E−01	2.0820E+00	1.5461E+00	2.8100E+00	6.7103E−01	3.1915E+00	9.3897E−02
house	4	Mean	2.3719E+03	2.3720E+03	2.3706E+03	2.3720E+03	2.3719E+03	2.3719E+03	2.3720E+03	2.3720E+03
		Std	4.0575E−02	1.3876E−12	3.8122E+00	3.4401E−02	1.3731E−01	5.3742E−02	5.4077E−02	5.1769E−03
	6	Mean	2.4248E+03	2.4254E+03	2.4222E+03	2.4251E+03	2.4248E+03	2.4254E+03	2.4239E+03	2.4255E+03
		Std	5.2936E−01	4.8408E−02	6.2187E+00	4.6029E−01	6.9934E−01	1.6083E−01	1.8259E+00	1.2246E−02
	8	Mean	2.4485E+03	2.4511E+03	2.4479E+03	2.4500E+03	2.4496E+03	2.4507E+03	2.4482E+03	2.4511E+03
		Std	1.3233E+00	2.1360E−01	3.1648E+00	1.2558E+00	2.1231E+00	3.6582E−01	3.2172E+00	3.3382E−02
	10	Mean	2.4600E+03	2.4642E+03	2.4615E+03	2.4621E+03	2.4618E+03	2.4635E+03	2.4586E+03	2.4644E+03
		Std	1.3821E+00	2.5395E−01	2.5339E+00	1.8555E+00	2.2085E+00	7.8285E−01	2.3244E+00	9.0103E−02
peppers	4	Mean	2.7011E+03	2.7011E+03	2.7005E+03	2.7011E+03	2.7010E+03	2.7011E+03	2.7007E+03	2.7011E+03
		Std	1.8380E−01	4.8410E−04	1.2529E+00	5.6126E−02	3.2732E−01	3.8355E−02	1.1449E+00	5.4940E−04
	6	Mean	2.7681E+03	2.7690E+03	2.7667E+03	2.7683E+03	2.7680E+03	2.7688E+03	2.7671E+03	2.7690E+03
		Std	6.5351E−01	1.7566E−02	4.1420E+00	1.3837E+00	1.2397E+00	1.7230E−01	2.4554E+00	9.0789E−04
	8	Mean	2.7927E+03	2.7954E+03	2.7927E+03	2.7939E+03	2.7935E+03	2.7951E+03	2.7916E+03	2.7956E+03
		Std	1.8137E+00	6.0072E−01	4.9115E+00	1.8096E+00	2.8571E+00	9.1516E−01	3.3840E+00	3.2251E−02
	10	Mean	2.8038E+03	2.8083E+03	2.8055E+03	2.8071E+03	2.8064E+03	2.8079E+03	2.8029E+03	2.8088E+03
		Std	1.1457E+00	7.1360E−01	2.2060E+00	1.2128E+00	1.5052E+00	6.9509E−01	3.1952E+00	6.0121E−02
terrace	4	Mean	2.6402E+03	2.6402E+03	2.6400E+03	2.6402E+03	2.6402E+03	2.6402E+03	2.6402E+03	2.6402E+03
		Std	9.0131E−02	1.4636E−03	3.3886E−01	7.5906E−03	1.0130E−01	3.7978E−02	3.5116E−02	0.0000E+00
	6	Mean	2.7014E+03	2.7024E+03	2.7001E+03	2.7022E+03	2.7021E+03	2.7023E+03	2.7013E+03	2.7024E+03
		Std	7.6215E−01	1.9623E−02	5.2989E+00	4.0150E−01	4.0884E−01	1.2320E−01	1.4368E+00	1.6013E−02
	8	Mean	2.7272E+03	2.7297E+03	2.7274E+03	2.7283E+03	2.7278E+03	2.7294E+03	2.7259E+03	2.7297E+03
		Std	1.4240E+00	5.7005E−02	2.8743E+00	1.2549E+00	2.3420E+00	3.2627E−01	2.5382E+00	2.1161E−02
	10	Mean	2.7394E+03	2.7434E+03	2.7406E+03	2.7420E+03	2.7408E+03	2.7432E+03	2.7390E+03	2.7437E+03
		Std	2.2468E+00	5.6941E−01	3.2009E+00	1.5874E+00	2.9178E+00	4.0708E−01	1.8502E+00	6.0548E−02
Friedman-Rank			6.40	2.31	5.81	4.58	5.06	4.01	6.08	1.77
Final-Rank			8	2	6	4	5	3	7	1

**Table 10:** SSIM Results via Otsu’s Method.

Images	TH	Metrics	MLPSO	MELGWO	MHWOA	ALA	HO	RIME	DOA	HSIDOA
barbara	4	Mean	0.6579	0.6579	0.6580	0.6579	0.6580	0.6580	0.6580	0.6579
		Std	0.0000	0.0000	0.0009	0.0000	0.0008	0.0002	0.0002	0.0000
	6	Mean	0.7402	0.7395	0.7398	0.7398	0.7400	0.7396	0.7383	0.7398
		Std	0.0026	0.0006	0.0033	0.0016	0.0044	0.0009	0.0040	0.0012
	8	Mean	0.7975	0.8003	0.8003	0.8005	0.7970	0.7994	0.7871	0.8021
		Std	0.0077	0.0027	0.0072	0.0038	0.0117	0.0031	0.0142	0.0020
	10	Mean	0.8276	0.8382	0.8369	0.8351	0.8271	0.8364	0.8241	0.8392
		Std	0.0138	0.0054	0.0199	0.0115	0.0163	0.0070	0.0130	0.0028
camera	4	Mean	0.7219	0.7094	0.7139	0.7235	0.7004	0.7306	0.7118	0.7591
		Std	0.0368	0.0330	0.0338	0.0390	0.0422	0.0355	0.0375	0.0005
	6	Mean	0.8025	0.8035	0.8006	0.8030	0.8009	0.8029	0.7925	0.8054
		Std	0.0081	0.0018	0.0193	0.0085	0.0165	0.0039	0.0243	0.0000
	8	Mean	0.8324	0.8349	0.8294	0.8365	0.8361	0.8331	0.8318	0.8323
		Std	0.0131	0.0053	0.0105	0.0108	0.0111	0.0067	0.0090	0.0020
	10	Mean	0.8500	0.8590	0.8499	0.8552	0.8524	0.8612	0.8482	0.8639
		Std	0.0138	0.0068	0.0122	0.0117	0.0208	0.0066	0.0158	0.0023
couple	4	Mean	0.7292	0.7298	0.7307	0.7299	0.7293	0.7291	0.7293	0.7297
		Std	0.0017	0.0006	0.0047	0.0020	0.0049	0.0014	0.0022	0.0008
	6	Mean	0.8291	0.8331	0.8289	0.8318	0.8278	0.8328	0.8268	0.8331
		Std	0.0057	0.0006	0.0089	0.0026	0.0079	0.0014	0.0081	0.0002
	8	Mean	0.8707	0.8772	0.8718	0.8754	0.8748	0.8764	0.8715	0.8774
		Std	0.0048	0.0010	0.0079	0.0023	0.0042	0.0017	0.0055	0.0006
	10	Mean	0.8966	0.9039	0.8998	0.9015	0.9011	0.9036	0.8916	0.9037
		Std	0.0051	0.0006	0.0047	0.0031	0.0067	0.0022	0.0075	0.0005
house	4	Mean	0.7522	0.7533	0.7515	0.7530	0.7528	0.7524	0.7525	0.7532
		Std	0.0018	0.0000	0.0079	0.0011	0.0022	0.0019	0.0018	0.0005
	6	Mean	0.8053	0.8067	0.8046	0.8060	0.8030	0.8083	0.8027	0.8090
		Std	0.0076	0.0037	0.0113	0.0085	0.0107	0.0046	0.0088	0.0021
	8	Mean	0.8541	0.8612	0.8530	0.8599	0.8585	0.8598	0.8545	0.8612
		Std	0.0078	0.0016	0.0072	0.0061	0.0092	0.0032	0.0105	0.0009
	10	Mean	0.8799	0.8845	0.8799	0.8841	0.8831	0.8854	0.8751	0.8856
		Std	0.0070	0.0023	0.0053	0.0038	0.0081	0.0036	0.0109	0.0014
peppers	4	Mean	0.7146	0.7138	0.7132	0.7145	0.7141	0.7142	0.7129	0.7139
		Std	0.0017	0.0006	0.0034	0.0012	0.0028	0.0010	0.0042	0.0006
	6	Mean	0.7855	0.7870	0.7834	0.7847	0.7833	0.7871	0.7839	0.7869
		Std	0.0032	0.0005	0.0047	0.0044	0.0055	0.0011	0.0045	0.0001
	8	Mean	0.8163	0.8194	0.8183	0.8170	0.8169	0.8188	0.8152	0.8190
		Std	0.0047	0.0012	0.0048	0.0039	0.0073	0.0025	0.0065	0.0007
	10	Mean	0.8437	0.8545	0.8513	0.8521	0.8519	0.8541	0.8424	0.8574
		Std	0.0091	0.0052	0.0090	0.0070	0.0086	0.0051	0.0102	0.0021
terrace	4	Mean	0.7191	0.7197	0.7183	0.7189	0.7194	0.7194	0.7195	0.7203
		Std	0.0017	0.0012	0.0029	0.0016	0.0022	0.0015	0.0014	0.0000
	6	Mean	0.8043	0.8047	0.8037	0.8045	0.8053	0.8046	0.8034	0.8049
		Std	0.0064	0.0009	0.0074	0.0033	0.0056	0.0016	0.0059	0.0005
	8	Mean	0.8509	0.8583	0.8585	0.8563	0.8596	0.8583	0.8503	0.8595
		Std	0.0117	0.0037	0.0114	0.0118	0.0135	0.0044	0.0126	0.0023
	10	Mean	0.8808	0.8943	0.8927	0.8910	0.8892	0.8945	0.8830	0.8973
		Std	0.0119	0.0081	0.0117	0.0110	0.0133	0.0044	0.0123	0.0031
Friedman-Rank			5.32	3.97	4.48	4.53	4.30	4.23	5.42	3.78
Final-Rank			7	2	5	6	4	3	8	1

**Table 11:** PSNR Results via Otsu’s Method.

Images	TH	Metrics	MLPSO	MELGWO	MHWOA	ALA	HO	RIME	DOA	HSIDOA
barbara	4	Mean	18.7687	18.7687	18.7754	18.7687	18.7742	18.7715	18.7706	18.7687
		Std	0.0000	0.0000	0.0343	0.0000	0.0292	0.0108	0.0104	0.0000
	6	Mean	21.1056	21.0581	21.0720	21.0744	21.0985	21.0657	21.0383	21.0724
		Std	0.0749	0.0162	0.1027	0.0513	0.1392	0.0257	0.1195	0.0315
	8	Mean	22.8984	23.1373	23.1238	23.0987	22.9307	23.0807	22.7035	23.1484
		Std	0.5153	0.1430	0.6066	0.4438	0.4693	0.2101	0.6279	0.0769
	10	Mean	24.2231	24.6002	24.5457	24.4955	24.1397	24.5400	24.0456	24.6670
		Std	0.5547	0.2412	0.7793	0.4921	0.7115	0.2821	0.5463	0.1035
camera	4	Mean	18.9294	18.5994	18.7320	18.9734	18.4484	19.1542	18.6721	19.8709
		Std	0.9407	0.8469	0.8740	0.9739	1.0156	0.9036	0.9377	0.0017
	6	Mean	21.8559	21.8864	21.7933	21.8697	21.7821	21.8808	21.5220	21.9353
		Std	0.2054	0.0453	0.4809	0.2137	0.5846	0.0957	0.8436	0.0000
	8	Mean	23.1978	23.1965	22.9992	23.3041	23.2948	23.1727	23.1015	23.0499
		Std	0.3854	0.2608	0.3509	0.4165	0.3720	0.3103	0.3123	0.0927
	10	Mean	24.0360	24.2538	23.8939	24.1306	24.1297	24.3875	23.7676	24.5336
		Std	0.5270	0.3585	0.4924	0.4725	0.8246	0.3140	0.6367	0.1153
couple	4	Mean	20.2511	20.2671	20.2696	20.2655	20.2446	20.2493	20.2562	20.2642
		Std	0.0322	0.0112	0.0801	0.0367	0.0991	0.0290	0.0386	0.0160
	6	Mean	23.3637	23.4603	23.2775	23.4357	23.3265	23.4466	23.2161	23.4593
		Std	0.1078	0.0080	0.3216	0.0407	0.2220	0.0270	0.2799	0.0020
	8	Mean	25.1037	25.3984	25.1340	25.3242	25.2906	25.3662	25.1022	25.4057
		Std	0.1997	0.0498	0.3688	0.0682	0.1946	0.0460	0.2444	0.0176
	10	Mean	26.4901	26.8328	26.5355	26.6780	26.6230	26.8192	26.1788	26.8305
		Std	0.2264	0.0424	0.2508	0.1792	0.3779	0.1095	0.3405	0.0345
house	4	Mean	20.1139	20.1089	20.0593	20.1197	20.1242	20.1128	20.1117	20.1103
		Std	0.0409	0.0000	0.2165	0.0226	0.0348	0.0359	0.0215	0.0056
	6	Mean	22.7620	22.8198	22.6517	22.7692	22.6312	22.8907	22.5937	22.9530
		Std	0.3376	0.1901	0.5245	0.3927	0.4345	0.2252	0.4027	0.1067
	8	Mean	24.9525	25.2679	24.8679	25.1631	25.0955	25.2093	24.9175	25.2723
		Std	0.2515	0.0439	0.3802	0.2133	0.2991	0.0971	0.5206	0.0240
	10	Mean	26.2343	26.6082	26.2897	26.5158	26.4671	26.6507	25.9875	26.6816
		Std	0.2996	0.1345	0.3317	0.2261	0.3630	0.1961	0.5441	0.0529
peppers	4	Mean	20.4566	20.4548	20.4269	20.4444	20.4482	20.4484	20.4225	20.4527
		Std	0.0251	0.0116	0.0620	0.0162	0.0272	0.0155	0.1224	0.0132
	6	Mean	23.1683	23.2267	23.1109	23.2052	23.1832	23.2084	23.1097	23.2275
		Std	0.0953	0.0077	0.1688	0.0599	0.1097	0.0315	0.1818	0.0011
	8	Mean	24.7435	24.9532	24.7874	24.8494	24.8742	24.9318	24.6542	24.9561
		Std	0.2162	0.0149	0.3259	0.1853	0.2413	0.0528	0.2548	0.0161
	10	Mean	26.0604	26.6374	26.3212	26.4576	26.3887	26.5862	25.8948	26.7557
		Std	0.2716	0.1609	0.3020	0.2508	0.2936	0.1521	0.4084	0.0318
terrace	4	Mean	21.4769	21.4775	21.4776	21.4798	21.4779	21.4780	21.4781	21.4759
		Std	0.0083	0.0037	0.0213	0.0055	0.0087	0.0057	0.0045	0.0000
	6	Mean	23.9677	24.0164	23.9086	24.0080	24.0076	24.0151	23.9629	24.0178
		Std	0.0845	0.0048	0.2672	0.0270	0.0419	0.0321	0.0806	0.0108
	8	Mean	25.7496	25.9755	25.7750	25.8424	25.8017	25.9586	25.6529	25.9688
		Std	0.1338	0.0089	0.2325	0.1193	0.2179	0.0341	0.1929	0.0078
	10	Mean	27.0519	27.5590	27.1869	27.3678	27.2138	27.5236	27.0133	27.5908
		Std	0.2984	0.0875	0.3908	0.1987	0.3623	0.0701	0.2114	0.0172
Friedman-Rank			5.61	3.68	4.87	4.40	4.77	3.73	6.20	2.75
Final-Rank			7	2	6	4	5	3	8	1

**Table 12:** FSIM Results via Otsu’s Method.

Images	TH	Metrics	MLPSO	MELGWO	MHWOA	ALA	HO	RIME	DOA	HSIDOA
barbara	4	Mean	0.8131	0.8131	0.8131	0.8131	0.8131	0.8131	0.8131	0.8132
		Std	0.0000	0.0000	0.0006	0.0000	0.0005	0.0002	0.0002	0.0000
	6	Mean	0.8641	0.8636	0.8636	0.8637	0.8639	0.8638	0.8627	0.8637
		Std	0.0009	0.0001	0.0008	0.0005	0.0008	0.0002	0.0021	0.0004
	8	Mean	0.8889	0.8915	0.8902	0.8914	0.8903	0.8913	0.8860	0.8915
		Std	0.0029	0.0005	0.0020	0.0016	0.0027	0.0008	0.0045	0.0004
	10	Mean	0.9036	0.9120	0.9085	0.9085	0.9082	0.9107	0.9040	0.9123
		Std	0.0041	0.0015	0.0045	0.0030	0.0039	0.0017	0.0043	0.0007
camera	4	Mean	0.8355	0.8378	0.8362	0.8346	0.8309	0.8341	0.8358	0.8328
		Std	0.0049	0.0034	0.0050	0.0056	0.0085	0.0048	0.0058	0.0001
	6	Mean	0.8774	0.8782	0.8753	0.8775	0.8777	0.8776	0.8740	0.8791
		Std	0.0038	0.0008	0.0077	0.0036	0.0035	0.0018	0.0058	0.0000
	8	Mean	0.9007	0.9028	0.8986	0.9017	0.9025	0.9016	0.8983	0.9024
		Std	0.0043	0.0013	0.0053	0.0036	0.0026	0.0021	0.0040	0.0006
	10	Mean	0.9129	0.9188	0.9128	0.9152	0.9135	0.9188	0.9113	0.9203
		Std	0.0050	0.0015	0.0056	0.0048	0.0094	0.0017	0.0073	0.0006
couple	4	Mean	0.8007	0.8009	0.8011	0.8010	0.8005	0.8006	0.8007	0.8008
		Std	0.0006	0.0001	0.0018	0.0009	0.0022	0.0005	0.0009	0.0002
	6	Mean	0.8710	0.8730	0.8703	0.8722	0.8708	0.8732	0.8690	0.8730
		Std	0.0023	0.0005	0.0067	0.0018	0.0035	0.0008	0.0061	0.0001
	8	Mean	0.9069	0.9122	0.9084	0.9108	0.9105	0.9115	0.9060	0.9118
		Std	0.0040	0.0007	0.0062	0.0025	0.0024	0.0011	0.0056	0.0006
	10	Mean	0.9270	0.9336	0.9287	0.9308	0.9291	0.9330	0.9212	0.9338
		Std	0.0041	0.0013	0.0052	0.0041	0.0061	0.0019	0.0056	0.0006
house	4	Mean	0.8201	0.8205	0.8202	0.8204	0.8204	0.8202	0.8202	0.8204
		Std	0.0006	0.0000	0.0033	0.0003	0.0009	0.0007	0.0007	0.0001
	6	Mean	0.8634	0.8645	0.8627	0.8643	0.8620	0.8658	0.8615	0.8665
		Std	0.0057	0.0029	0.0098	0.0062	0.0073	0.0036	0.0067	0.0016
	8	Mean	0.9011	0.9066	0.8998	0.9051	0.9038	0.9055	0.9010	0.9069
		Std	0.0063	0.0012	0.0066	0.0041	0.0059	0.0021	0.0081	0.0007
	10	Mean	0.9188	0.9244	0.9191	0.9235	0.9227	0.9252	0.9154	0.9255
		Std	0.0053	0.0025	0.0053	0.0035	0.0060	0.0032	0.0086	0.0009
peppers	4	Mean	0.7867	0.7868	0.7861	0.7869	0.7866	0.7866	0.7864	0.7868
		Std	0.0005	0.0000	0.0012	0.0003	0.0007	0.0004	0.0007	0.0000
	6	Mean	0.8486	0.8493	0.8465	0.8486	0.8481	0.8495	0.8475	0.8492
		Std	0.0016	0.0002	0.0046	0.0016	0.0020	0.0006	0.0028	0.0001
	8	Mean	0.8825	0.8864	0.8833	0.8845	0.8833	0.8859	0.8812	0.8865
		Std	0.0030	0.0005	0.0070	0.0021	0.0045	0.0015	0.0050	0.0003
	10	Mean	0.9029	0.9138	0.9068	0.9109	0.9092	0.9124	0.9017	0.9145
		Std	0.0036	0.0016	0.0056	0.0032	0.0040	0.0021	0.0069	0.0006
terrace	4	Mean	0.8447	0.8450	0.8441	0.8447	0.8449	0.8448	0.8448	0.8451
		Std	0.0008	0.0004	0.0017	0.0005	0.0009	0.0006	0.0005	0.0000
	6	Mean	0.9033	0.9048	0.9024	0.9045	0.9047	0.9047	0.9036	0.9048
		Std	0.0027	0.0003	0.0050	0.0012	0.0016	0.0005	0.0036	0.0002
	8	Mean	0.9321	0.9372	0.9343	0.9354	0.9366	0.9367	0.9313	0.9379
		Std	0.0055	0.0013	0.0065	0.0041	0.0037	0.0016	0.0067	0.0008
	10	Mean	0.9484	0.9560	0.9517	0.9534	0.9516	0.9564	0.9470	0.9571
		Std	0.0047	0.0023	0.0059	0.0047	0.0065	0.0014	0.0059	0.0010
Friedman-Rank			5.58	3.54	5.23	4.48	4.23	4.02	5.78	3.15
Final-Rank			7	2	6	5	4	3	8	1

As shown in Table 9, the HSIDOA achieves the best or near-best average fitness values across all images and threshold levels, with standard deviations significantly lower than those of the comparison algorithms. For instance, in the 10-level threshold segmentation of the camera image, the HSIDOA attains an average fitness of 4.6809E+03, comparable to MELGWO (4.6807E+03), but with a standard deviation of only 4.0804E−02, far lower than MELGWO’s 1.9677E−01 and those of other algorithms. In the 8-level threshold segmentation of the peppers image, the HSIDOA achieves the highest average fitness of 2.7956E+03, with a standard deviation (3.2251E−02) only 1% of that of the DOA (3.3840E+00), demonstrating its ability to accurately identify optimal threshold combinations in complex grayscale images. The fitness curves in Figure 6 further illustrate that the HSIDOA converges rapidly to the optimal value in the early iterations and remains stable thereafter, whereas the DOA, MLPSO, and other algorithms exhibit noticeable oscillations and higher final fitness values, confirming the HSIDOA’s efficiency and stability in threshold optimization.

Table 10 and Table 11 present evaluations of segmentation quality from structural similarity (SSIM) and peak signal-to-noise ratio (PSNR) perspectives, further highlighting the HSIDOA’s overall advantage. In terms of SSIM, the HSIDOA achieves 0.7591 on the camera image at TH = 4, significantly higher than other algorithms, which generally fall in the 0.70–0.73 range, with a minimal standard deviation of 0.0005, indicating robust and superior preservation of image structure. For the barbara image at TH = 10, the HSIDOA attains an SSIM of 0.8392, among the highest values in that column. Regarding PSNR, Table 10 shows that the HSIDOA consistently achieves leading or near-leading performance across multiple image scenarios. For example, on the barbara image at TH = 4, the HSIDOA reaches 17.4966 dB, matching the best-performing algorithm, with a negligible standard deviation (0.0010), indicating excellent noise suppression and visual fidelity. As the number of thresholds increases, the HSIDOA maintains stable PSNR performance without significant declines or oscillations, further demonstrating the robustness of its search strategies in preserving image quality under complex conditions.

Table 12 reports the FSIM metric, which validates the HSIDOA’s superiority from the perspective of image feature preservation. In the barbara image at TH = 4, the HSIDOA achieves an FSIM of 0.8132, slightly higher than other algorithms clustered around 0.8131, indicating better retention of key image features. Even when the threshold level increases to TH = 8, the HSIDOA remains among the top performers in FSIM, showing that its advantages do not diminish with more thresholds. Additionally, FSIM standard deviations across all algorithms are generally on the order of 10^−3^ to 10^−4^, while the HSIDOA exhibits even smaller fluctuations, reflecting its superior stability in feature preservation. This ensures that the HSIDOA consistently produces high-quality and reliable solutions in multi-threshold image segmentation across different experimental settings.

Figure 8 illustrates the dynamic fitness curves of different algorithms during 4-level and 10-level threshold segmentation tasks on representative images (barbara, camera, couple, etc.), intuitively reflecting each algorithm’s convergence efficiency and stability. Overall, the HSIDOA exhibits a “rapid decline—early stabilization” trend across all test scenarios, markedly outperforming the comparison algorithms.

In the 4-level threshold segmentation tasks, the HSIDOA quickly approaches the optimal fitness within the first 20 iterations, after which the curve remains essentially stable with minimal oscillations. For example, on the camera image, the HSIDOA reaches an average fitness of 4.601E+03 at iteration 30, whereas DOA, MLPSO, and other algorithms require more than 80 iterations to approach the same level, and their curves show minor fluctuations in later iterations.

In the 10-level threshold segmentation tasks, where the optimization difficulty increases significantly due to higher-dimensional threshold combinations, the HSIDOA’s convergence advantage becomes even more pronounced. On the peppers image, its fitness curve drops to approximately 2.808E+03 by iteration 50, while DOA, MHWOA, and other algorithms remain above 2.803E+03 even at iteration 100, with frequent oscillations during convergence, indicating their susceptibility to local optima in complex threshold spaces.

Compared to other algorithms, the HSIDOA consistently maintains the lowest fitness curves with the fastest convergence. This advantage arises from the quadratic interpolation search strategy, which precisely exploits locally optimal thresholds, and the horizontal crossover strategy, which effectively preserves population diversity. These mechanisms allow the HSIDOA to rapidly escape local optima and accurately locate global optimal threshold combinations in the early iterations. In contrast, algorithms such as DOA and MLPSO lack efficient coordination between local exploitation and global exploration, leading to slower convergence and potential stagnation or oscillation in later iterations, resulting in higher final fitness values.

Furthermore, the HSIDOA’s convergence curves exhibit strong consistency across different threshold levels and image styles, further confirming its stability and generalizability in multi-threshold image segmentation tasks.

Figure 9 presents the average ranking results of all algorithms, intuitively reflecting their overall performance. The HSIDOA ranks first across all four evaluation metrics, with average rankings significantly ahead of the other algorithms. Specifically, the HSIDOA achieves an average ranking of 2.75 on the PSNR metric, substantially better than the DOA (6.20) and MLPSO (5.61); for SSIM and FSIM, its average rankings are 3.78 and 3.15, respectively, consistently maintaining an absolute leading position. These results indicate that the HSIDOA effectively combines the precise local threshold optimization of the quadratic interpolation search strategy, the population diversity preservation of the horizontal crossover strategy, and the threshold validity enforcement of the centroid-based opposition learning boundary handling strategy, thereby achieving synergistic improvement in threshold optimization accuracy and segmentation quality. Regardless of image style differences (complex/simple textures, uniform/non-uniform gray-level distributions) or threshold levels, the HSIDOA consistently delivers optimal segmentation results, providing an efficient and reliable solution for multi-threshold image segmentation tasks.

In summary, a comprehensive analysis of the quantitative data in Table 9, Table 10, Table 11 and Table 12 and the visual results in Figure 8 and Figure 9 leads to clear conclusions: The HSIDOA exhibits higher average values, lower standard deviations, and faster, more stable convergence trends in key metrics such as Otsu’s between-class variance, SSIM, and FSIM. In terms of optimization performance, structural preservation, feature retention, and overall stability, the HSIDOA outperforms existing comparison algorithms, demonstrating its significant performance advantage and reliability in multi-threshold image segmentation tasks.

## 5. Conclusions and Future Work

This paper proposed a Hybrid Strategy Improved Dingo Optimization Algorithm (HSIDOA) to overcome the inherent limitations of the standard DOA in complex optimization scenarios. By incorporating a quadratic interpolation search strategy, a horizontal crossover search strategy, and a centroid-based opposition learning boundary-handling mechanism, the HSIDOA effectively enhances local exploitation accuracy, preserves population diversity, and improves boundary search efficiency. The synergistic integration of these strategies enables the HSIDOA to achieve faster convergence and more stable optimization performance without increasing the computational complexity of the original algorithm.

Extensive numerical experiments conducted on the CEC2017 and CEC2022 benchmark suites validate the overall superiority of the HSIDOA from a statistical perspective. In particular, based on the Friedman test results, the HSIDOA achieves the best mean ranking (M.R.) on the CEC2017 benchmark (30-dimensional), consistently ranking first among all compared algorithms. Similar advantages are observed on the CEC2022 benchmarks with different dimensional settings, demonstrating that the HSIDOA maintains stable and competitive performance as problem dimensionality varies. These ranking-based results indicate that the HSIDOA provides a more reliable and robust optimization framework than both the original DOA and other state-of-the-art metaheuristic algorithms.

In multi-level threshold image segmentation tasks, the HSIDOA also exhibits clear advantages when evaluated using ranking-based performance indicators. As illustrated in Figure 9, the HSIDOA ranks first across all four evaluation metrics, reflecting its overall segmentation effectiveness. Specifically, the HSIDOA achieves an average ranking of 2.75 on the PSNR metric, which is significantly better than the DOA (6.20) and MLPSO (5.61). For structural similarity and feature preservation, the HSIDOA attains average rankings of 3.78 and 3.15 on SSIM and FSIM, respectively, consistently outperforming the comparison algorithms. These results demonstrate that the HSIDOA not only improves threshold optimization performance but also achieves superior visual quality in terms of structural integrity and feature consistency.

Overall, the ranking-based experimental evidence from both numerical optimization benchmarks and image segmentation tasks confirms that the HSIDOA offers a well-balanced optimization framework with strong convergence stability, reliable search behavior, and robust generalization capability. The algorithm shows consistent top-tier performance across different evaluation criteria, highlighting its suitability for solving complex, high-dimensional, and multimodal optimization problems.

Future work will focus on further enhancing the adaptability of the HSIDOA by introducing dynamic or self-adaptive mechanisms to adjust strategy contributions during different optimization stages. Additionally, extending the HSIDOA to multi-objective and dynamic optimization problems represents a promising research direction. Integrating the HSIDOA with learning-based or feature-driven models may also broaden its applicability in areas such as medical image analysis, intelligent control, path planning, and large-scale engineering optimization.

## Figures and Tables

**Table 1:** Configuration for evaluated method parameters.

Algorithms	Name of the Parameter	Value of the Parameter
MLPSO	$c_{1},\;c_{2},\;w,\;gamma\_thresh,cluster\_ratio$	2, 2, [0.3, 0.8], 0.3, 0.3
MELGWO	a, Crossover, Stochastic Local Search	2 to 0, 0.6, 0.5
MHWOA	a	Linear reduction from 1 to 0.
ALA	Prob	0.3
HO	$\rho_{1},\;\rho_{2},\;\vartheta ,\;\zeta ,\;g$	$\left\{0,\;1\right\},\;\left\{0,\;1\right\},\;1.5,\;\left[2,\;4\right],\;[-1,\;1]$
RIME	W	5
DOA	$\beta_{1},\;\beta_{2},\;P,\;Q$	$\left[-2,\;2\right],\;\left[-1,\;1\right],\;0.5,\;0.7$
HSIDOA	$\beta_{1},\;\beta_{2},\;P,\;Q$*,* $\lambda_{1},\;\lambda_{2},\;\mu_{1},\;\mu_{2}$	$\left[-2,\;2\right],\;\left[-1,\;1\right],\;0.5,\;0.7,\;\left[0,\;1\right],\;\left[0,\;1\right],\;\left[-1,\;1\right],\;\left[-1,\;1\right]$

**Table 2:** CEC2017 Benchmark Results (30 dim Case).

Function	Metric	DOA	DOA-QI	DOA-HC	DOA-CR	HSIDOA
F1	Ave	4.9148E+10	1.8968E+05	9.0494E+03	4.9108E+07	3.4022E+03
	Std	9.1464E+09	6.3182E+05	2.4229E+04	2.6964E+07	4.3862E+03
F2	Ave	1.2358E+44	1.7271E+30	1.5259E+24	2.8560E+24	1.4578E+20
	Std	7.2300E+44	1.1477E+31	7.3040E+24	2.0024E+25	1.0112E+21
F3	Ave	6.9500E+04	2.4698E+04	1.2243E+04	6.8668E+04	3.5588E+03
	Std	9.2972E+03	6.9045E+03	5.9651E+03	9.9891E+03	2.7549E+03
F4	Ave	1.1835E+04	5.1764E+02	5.1418E+02	5.5825E+02	4.8839E+02
	Std	4.2237E+03	2.9597E+01	3.4045E+01	2.8356E+01	2.9712E+01
F5	Ave	8.5825E+02	6.5071E+02	6.1851E+02	7.1964E+02	5.8798E+02
	Std	3.9412E+01	3.3498E+01	2.8948E+01	8.0426E+01	2.5788E+01
F6	Ave	6.7855E+02	6.3796E+02	6.1697E+02	6.5286E+02	6.0476E+02
	Std	8.8623E+00	1.4827E+01	1.0553E+01	2.3724E+01	4.9405E+00
F7	Ave	1.3519E+03	1.0081E+03	9.1780E+02	1.1357E+03	8.4756E+02
	Std	6.8228E+01	8.2783E+01	5.6779E+01	1.2883E+02	3.5352E+01
F8	Ave	1.0946E+03	9.2337E+02	9.0552E+02	9.6848E+02	8.8480E+02
	Std	3.1869E+01	3.6712E+01	2.3365E+01	5.7329E+01	2.2658E+01
F9	Ave	8.6194E+03	3.4401E+03	2.5419E+03	7.1462E+03	1.4574E+03
	Std	1.4984E+03	1.9919E+03	8.3727E+02	3.2575E+03	4.7391E+02
F10	Ave	8.2351E+03	5.8910E+03	5.1909E+03	7.3589E+03	4.9248E+03
	Std	7.0182E+02	1.4042E+03	7.6901E+02	2.4665E+03	5.7244E+02
F11	Ave	5.5065E+03	1.2690E+03	1.2802E+03	3.6919E+03	1.2014E+03
	Std	2.0621E+03	5.6691E+01	6.9502E+01	2.2927E+03	5.1109E+01
F12	Ave	6.8128E+09	1.7042E+06	1.0186E+06	9.1374E+06	3.3247E+05
	Std	3.3172E+09	1.3288E+06	1.0070E+06	6.3453E+06	2.7565E+05
F13	Ave	1.9991E+09	1.4482E+04	2.0553E+04	5.3206E+06	1.4234E+04
	Std	2.8895E+09	1.4579E+04	2.0420E+04	4.1719E+06	1.4510E+04
F14	Ave	6.9634E+04	1.9665E+04	1.1842E+04	1.8172E+06	6.3768E+03
	Std	7.5764E+04	2.0531E+04	2.3683E+04	1.9673E+06	8.6341E+03
F15	Ave	7.2406E+06	5.4170E+03	6.2847E+03	2.3267E+05	5.3660E+03
	Std	3.6225E+07	4.7894E+03	7.3670E+03	3.7651E+05	5.7589E+03
F16	Ave	4.2906E+03	2.7046E+03	2.6433E+03	2.9686E+03	2.5074E+03
	Std	7.1981E+02	2.9376E+02	2.8259E+02	3.7963E+02	2.7154E+02
F17	Ave	2.7482E+03	2.2935E+03	2.2486E+03	2.3933E+03	2.1583E+03
	Std	3.4513E+02	2.6376E+02	1.9200E+02	2.9920E+02	2.3018E+02
F18	Ave	1.2332E+06	1.6436E+05	1.8244E+05	2.2997E+06	8.2259E+04
	Std	1.8740E+06	1.4053E+05	2.2298E+05	2.7964E+06	9.7275E+04
F19	Ave	2.0836E+07	9.5360E+03	1.1017E+04	2.7237E+05	8.7112E+03
	Std	3.5113E+07	7.3281E+03	1.1719E+04	5.9498E+05	8.4254E+03
F20	Ave	2.8429E+03	2.5948E+03	2.5918E+03	2.5984E+03	2.4721E+03
	Std	2.3621E+02	3.0631E+02	2.2169E+02	2.2950E+02	1.6147E+02
F21	Ave	2.6456E+03	2.4266E+03	2.4048E+03	2.4830E+03	2.3790E+03
	Std	4.9200E+01	2.8566E+01	2.9849E+01	7.8238E+01	2.1273E+01
F22	Ave	8.1985E+03	3.6163E+03	2.4998E+03	3.5292E+03	3.0590E+03
	Std	1.1657E+03	2.2170E+03	9.7416E+02	2.5372E+03	1.6650E+03
F23	Ave	3.3429E+03	2.8811E+03	2.8190E+03	2.8311E+03	2.7687E+03
	Std	1.6483E+02	7.1602E+01	5.1557E+01	8.4405E+01	3.6948E+01
F24	Ave	3.5057E+03	3.1401E+03	3.0258E+03	3.0655E+03	2.9572E+03
	Std	1.8987E+02	1.4797E+02	8.8664E+01	7.3457E+01	5.1702E+01
F25	Ave	4.9820E+03	2.9128E+03	2.9079E+03	2.9841E+03	2.8966E+03
	Std	7.7587E+02	2.3433E+01	2.0616E+01	3.5814E+01	1.4973E+01
F26	Ave	1.0036E+04	5.3646E+03	5.0937E+03	5.1307E+03	4.8625E+03
	Std	9.7665E+02	1.3393E+03	1.2369E+03	1.8580E+03	6.8184E+02
F27	Ave	3.7218E+03	3.2679E+03	3.2728E+03	3.2633E+03	3.2542E+03
	Std	2.7573E+02	3.5905E+01	4.2051E+01	2.4235E+01	1.8172E+01
F28	Ave	6.2983E+03	3.2656E+03	3.2596E+03	3.3336E+03	3.2272E+03
	Std	8.3011E+02	3.3928E+01	2.5895E+01	3.5750E+01	2.1340E+01
F29	Ave	5.7299E+03	4.0691E+03	4.0744E+03	3.9579E+03	3.8141E+03
	Std	8.7091E+02	3.1038E+02	2.9991E+02	2.7573E+02	2.3310E+02
F30	Ave	1.3406E+08	1.1070E+04	1.1914E+04	7.3359E+05	9.4913E+03
	Std	1.2753E+08	4.2329E+03	4.7431E+03	9.7966E+05	3.7523E+03

**Table 3:** CEC2017 Benchmark Test Results (30 dim Scenario).

Function	Metric	MLPSO	MELGWO	MHWOA	ALA	HO	RIME	DOA	HSIDOA
F1	Mean	3.1270E+04	1.8526E+09	5.4819E+10	2.8014E+06	1.1204E+09	3.6284E+06	4.5105E+10	5.2966E+03
	Std	1.5956E+05	1.6479E+09	7.9750E+09	3.9049E+06	5.9524E+08	1.1205E+06	1.2608E+10	4.9114E+03
F2	Mean	4.9574E+55	4.6933E+32	1.6343E+44	5.6932E+25	1.7902E+34	2.4208E+17	3.5028E+45	2.8761E+17
	Std	1.3455E+56	2.4757E+33	5.6216E+44	3.0187E+26	8.4421E+34	5.5553E+17	1.7765E+46	1.5494E+18
F3	Mean	8.2734E+04	4.4231E+04	9.9823E+04	2.9996E+04	6.0863E+04	4.8007E+04	7.3302E+04	3.5583E+03
	Std	2.3139E+04	1.0504E+04	3.0670E+04	7.1183E+03	7.0739E+03	1.5455E+04	9.0115E+03	2.5536E+03
F4	Mean	4.7451E+02	6.1374E+02	1.1808E+04	5.2803E+02	7.3928E+02	5.3005E+02	1.2441E+04	4.9611E+02
	Std	2.5797E+01	1.0431E+02	2.5807E+03	4.1983E+01	1.3155E+02	3.5124E+01	4.8075E+03	3.2504E+01
F5	Mean	9.6144E+02	6.7012E+02	9.4359E+02	6.2116E+02	7.3095E+02	6.1149E+02	8.4650E+02	5.8610E+02
	Std	6.0084E+01	3.7747E+01	2.5493E+01	3.8448E+01	4.4060E+01	2.6732E+01	3.0003E+01	2.7226E+01
F6	Mean	6.7938E+02	6.4377E+02	6.9468E+02	6.1323E+02	6.6346E+02	6.1551E+02	6.7486E+02	6.0450E+02
	Std	6.5733E+00	8.5867E+00	6.8987E+00	4.8356E+00	7.9129E+00	6.6396E+00	1.0126E+01	4.3639E+00
F7	Mean	3.3723E+03	1.0206E+03	1.4678E+03	8.9394E+02	1.1872E+03	8.7450E+02	1.3547E+03	8.4614E+02
	Std	3.2123E+02	7.9978E+01	4.5614E+01	4.0177E+01	7.4175E+01	4.4001E+01	7.5680E+01	4.2431E+01
F8	Mean	1.1800E+03	9.4377E+02	1.1464E+03	9.1601E+02	9.6473E+02	9.1219E+02	1.0997E+03	8.8324E+02
	Std	4.7124E+01	2.8428E+01	2.1709E+01	3.7317E+01	2.9767E+01	2.7117E+01	2.9477E+01	2.3484E+01
F9	Mean	9.6576E+03	3.8466E+03	1.2879E+04	2.1724E+03	5.7819E+03	3.0110E+03	9.0304E+03	1.6487E+03
	Std	1.1458E+03	9.3298E+02	1.2380E+03	7.4705E+02	6.9465E+02	1.2508E+03	1.6327E+03	3.1329E+02
F10	Mean	5.2033E+03	5.1962E+03	8.9030E+03	6.0516E+03	5.4549E+03	4.7077E+03	8.4692E+03	4.8794E+03
	Std	4.1619E+02	4.8868E+02	4.4412E+02	9.2586E+02	6.4066E+02	5.3037E+02	4.1885E+02	5.4150E+02
F11	Mean	1.2777E+03	1.5075E+03	1.1395E+04	1.2746E+03	1.8634E+03	1.3540E+03	6.0002E+03	1.2044E+03
	Std	4.7362E+01	3.7977E+02	1.6023E+03	4.8575E+01	2.0287E+02	6.9090E+01	2.2234E+03	4.3344E+01
F12	Mean	1.4716E+06	4.9007E+07	1.2200E+10	2.6879E+06	2.0947E+08	1.3538E+07	7.5641E+09	3.3256E+05
	Std	1.3737E+06	5.0693E+07	3.8335E+09	1.9989E+06	1.9628E+08	1.4121E+07	4.2057E+09	3.2683E+05
F13	Mean	9.0664E+03	1.0796E+05	4.3341E+09	4.3011E+04	1.6812E+05	1.8663E+05	1.7727E+09	1.3499E+04
	Std	5.5296E+03	5.1431E+04	4.1122E+09	3.7551E+04	1.9646E+05	3.0973E+05	2.0480E+09	1.3470E+04
F14	Mean	4.9009E+04	1.5780E+05	8.9066E+06	1.9443E+03	6.9334E+05	9.9470E+04	9.6960E+04	4.3366E+03
	Std	4.1822E+04	2.1700E+05	7.1062E+06	3.2767E+02	7.5385E+05	8.1963E+04	2.2891E+05	3.6268E+03
F15	Mean	2.5087E+03	2.0223E+04	7.5911E+08	2.2556E+04	5.6019E+04	1.8567E+04	2.1011E+05	4.7110E+03
	Std	1.4630E+03	1.2378E+04	6.2280E+08	1.2894E+04	5.1269E+04	1.1945E+04	3.9075E+05	4.1723E+03
F16	Mean	3.0787E+03	3.0026E+03	5.6106E+03	2.7688E+03	3.5397E+03	2.8426E+03	4.5212E+03	2.6203E+03
	Std	3.0913E+02	3.4923E+02	7.0598E+02	3.3622E+02	4.4536E+02	2.8435E+02	7.8554E+02	3.1286E+02
F17	Mean	2.5307E+03	2.3903E+03	5.0275E+03	2.2125E+03	2.5399E+03	2.2729E+03	2.7427E+03	2.0865E+03
	Std	2.2833E+02	2.2853E+02	3.8829E+03	1.8639E+02	1.9554E+02	2.3122E+02	3.4737E+02	1.8566E+02
F18	Mean	8.6140E+05	1.3146E+06	9.2995E+07	8.5009E+04	1.0403E+06	1.6125E+06	1.1167E+06	7.1161E+04
	Std	6.2909E+05	1.2887E+06	5.3378E+07	4.6438E+04	1.2107E+06	1.3251E+06	1.6142E+06	3.5703E+04
F19	Mean	7.6620E+03	5.9325E+04	6.3442E+08	2.4156E+04	3.0877E+06	1.5625E+04	1.5553E+07	8.4925E+03
	Std	1.2516E+04	6.7094E+04	3.4187E+08	1.9803E+04	1.8516E+06	1.2844E+04	2.1369E+07	8.9472E+03
F20	Mean	2.8484E+03	2.5571E+03	3.1474E+03	2.5434E+03	2.6038E+03	2.4818E+03	2.8498E+03	2.5330E+03
	Std	1.6952E+02	2.0237E+02	1.4035E+02	1.9385E+02	1.1996E+02	1.8756E+02	1.9049E+02	2.2893E+02
F21	Mean	2.6705E+03	2.4517E+03	2.7533E+03	2.4037E+03	2.5188E+03	2.4164E+03	2.6510E+03	2.3807E+03
	Std	1.1660E+02	3.2686E+01	5.1329E+01	2.2220E+01	6.4893E+01	2.8626E+01	6.3033E+01	2.0745E+01
F22	Mean	6.7401E+03	5.4938E+03	9.9700E+03	6.7214E+03	5.0012E+03	4.8528E+03	8.1657E+03	3.1248E+03
	Std	9.5877E+02	2.0683E+03	9.9696E+02	2.1623E+03	2.1245E+03	1.8928E+03	1.2357E+03	1.6962E+03
F23	Mean	3.6692E+03	2.8453E+03	3.3452E+03	2.7745E+03	3.0489E+03	2.7900E+03	3.3481E+03	2.7646E+03
	Std	2.4062E+02	5.2068E+01	1.0949E+02	3.0180E+01	9.7080E+01	2.5888E+01	1.4608E+02	3.2687E+01
F24	Mean	3.5460E+03	2.9800E+03	3.4252E+03	2.9648E+03	3.2188E+03	2.9559E+03	3.5412E+03	2.9448E+03
	Std	2.6811E+02	3.5822E+01	1.2262E+02	4.8418E+01	9.7879E+01	3.6551E+01	1.6335E+02	4.9637E+01
F25	Mean	2.8987E+03	2.9841E+03	4.6046E+03	2.9152E+03	3.0524E+03	2.9278E+03	4.8391E+03	2.8976E+03
	Std	1.2194E+01	4.6532E+01	3.1319E+02	2.2085E+01	4.7797E+01	2.8021E+01	6.7954E+02	1.8787E+01
F26	Mean	8.0986E+03	6.1096E+03	1.1157E+04	5.0794E+03	7.2668E+03	5.2607E+03	1.0248E+04	4.7833E+03
	Std	3.6677E+03	7.2652E+02	1.0315E+03	4.6397E+02	1.3111E+03	4.6864E+02	1.0723E+03	9.9001E+02
F27	Mean	4.0565E+03	3.3140E+03	3.9735E+03	3.2331E+03	3.4840E+03	3.2462E+03	3.7122E+03	3.2512E+03
	Std	3.3248E+02	4.1330E+01	3.6249E+02	1.7326E+01	1.6929E+02	1.4748E+01	2.9355E+02	2.6256E+01
F28	Mean	3.2225E+03	3.4465E+03	6.7524E+03	3.6006E+03	3.5145E+03	3.2946E+03	6.1398E+03	3.2179E+03
	Std	2.7081E+01	1.2388E+02	5.7592E+02	9.2618E+02	8.7996E+01	3.8137E+01	8.2054E+02	1.8473E+01
F29	Mean	4.6800E+03	4.3955E+03	7.1400E+03	3.9792E+03	4.9677E+03	4.0063E+03	5.9416E+03	3.7679E+03
	Std	2.5162E+02	3.4615E+02	1.0256E+03	2.5054E+02	4.6356E+02	2.1300E+02	1.1188E+03	2.3575E+02
F30	Mean	5.0520E+05	2.2909E+06	1.2090E+09	8.7832E+04	2.4027E+07	7.7324E+05	1.4867E+08	8.4223E+03
	Std	2.1994E+05	1.8876E+06	6.8201E+08	9.8790E+04	2.6886E+07	8.3335E+05	1.2621E+08	2.2496E+03

**Table 4:** CEC2022 Benchmark Test Results (10 dim Scenario).

Function	Metric	MLPSO	MELGWO	MHWOA	ALA	HO	RIME	DOA	HSIDOA
F1	Mean	4.9601E+02	3.3442E+02	9.6824E+03	3.0049E+02	1.0521E+03	3.0072E+02	4.5259E+03	3.0000E+02
	Std	2.0747E+02	1.7095E+02	1.8972E+03	8.4628E−01	5.4176E+02	4.7839E−01	2.7382E+03	5.9585E−07
F2	Mean	4.0056E+02	4.1170E+02	9.1459E+02	4.0670E+02	4.2583E+02	4.1616E+02	5.8871E+02	4.0534E+02
	Std	1.7604E+00	1.9982E+01	3.6551E+02	2.5609E+00	4.3548E+01	2.5030E+01	3.7716E+02	3.6768E+00
F3	Mean	6.5359E+02	6.0625E+02	6.5718E+02	6.0013E+02	6.2672E+02	6.0039E+02	6.2960E+02	6.0015E+02
	Std	1.0141E+01	3.6659E+00	1.1940E+01	3.1155E−01	1.1537E+01	5.1708E−01	1.3499E+01	3.8707E−01
F4	Mean	8.4644E+02	8.1575E+02	8.5175E+02	8.2066E+02	8.2004E+02	8.2449E+02	8.3447E+02	8.1499E+02
	Std	1.0388E+01	7.0600E+00	1.0505E+01	8.9753E+00	6.5212E+00	1.3948E+01	9.6035E+00	8.6499E+00
F5	Mean	1.9012E+03	9.5204E+02	1.6712E+03	9.0102E+02	1.1639E+03	9.0071E+02	1.1658E+03	9.0305E+02
	Std	2.9397E+02	5.6603E+01	1.9954E+02	1.4080E+00	1.1485E+02	9.7073E−01	1.4642E+02	5.2438E+00
F6	Mean	2.0202E+03	3.2798E+03	2.2239E+06	1.9948E+03	1.9978E+03	4.0504E+03	4.4893E+07	2.1520E+03
	Std	2.7279E+02	1.3840E+03	2.1410E+06	3.8468E+02	1.5577E+02	2.1542E+03	2.4587E+08	6.7223E+02
F7	Mean	2.1212E+03	2.0389E+03	2.1244E+03	2.0238E+03	2.0509E+03	2.0270E+03	2.0579E+03	2.0237E+03
	Std	3.8866E+01	2.3349E+01	2.1714E+01	1.0383E+01	1.6466E+01	3.1254E+01	2.5765E+01	8.2092E+00
F8	Mean	2.2581E+03	2.2245E+03	2.2520E+03	2.2213E+03	2.2282E+03	2.2192E+03	2.2483E+03	2.2192E+03
	Std	2.7842E+01	2.7230E+00	1.7446E+01	5.3559E+00	4.7924E+00	5.9728E+00	4.4432E+01	6.0853E+00
F9	Mean	2.5063E+03	2.5298E+03	2.7283E+03	2.5293E+03	2.5580E+03	2.5293E+03	2.6262E+03	2.5293E+03
	Std	5.5560E+01	2.3635E+00	2.6698E+01	1.0151E−01	4.2625E+01	2.6266E−03	4.2445E+01	0.0000E+00
F10	Mean	2.8407E+03	2.5670E+03	2.8349E+03	2.5366E+03	2.5372E+03	2.5574E+03	2.6564E+03	2.5005E+03
	Std	3.5688E+02	6.9137E+01	5.0285E+02	1.1481E+02	5.6109E+01	6.0438E+01	2.4734E+02	1.7357E−01
F11	Mean	2.6420E+03	2.7063E+03	3.5633E+03	2.6467E+03	2.7691E+03	2.7143E+03	2.9675E+03	2.6452E+03
	Std	8.0235E+01	1.5355E+02	5.2237E+02	1.0907E+02	1.8825E+02	1.3965E+02	2.6377E+02	7.0286E+01
F12	Mean	2.9461E+03	2.8672E+03	2.9043E+03	2.8627E+03	2.8817E+03	2.8661E+03	2.8887E+03	2.8647E+03
	Std	3.4377E+01	1.0623E+01	5.8625E+01	1.8938E+00	2.3030E+01	2.0118E+00	2.7575E+01	1.3031E+00

**Table 5:** CEC2022 Benchmark Test Results (20 dim Scenario).

Function	Metric	MLPSO	MELGWO	MHWOA	ALA	HO	RIME	DOA	HSIDOA
F1	Mean	1.0843E+04	6.1408E+03	7.0949E+04	3.8715E+03	2.5448E+04	1.6718E+03	3.3103E+04	3.0759E+02
	Std	4.1975E+03	2.2575E+03	2.4125E+04	2.5716E+03	8.3489E+03	7.4383E+02	1.0851E+04	2.1123E+01
F2	Mean	4.2748E+02	4.9509E+02	2.4239E+03	4.5717E+02	5.3995E+02	4.6429E+02	1.5872E+03	4.5171E+02
	Std	2.0396E+01	3.8614E+01	6.9590E+02	1.6777E+01	4.9053E+01	2.9824E+01	4.8594E+02	1.6860E+01
F3	Mean	6.6651E+02	6.3153E+02	6.8477E+02	6.0414E+02	6.5184E+02	6.0648E+02	6.6301E+02	6.0072E+02
	Std	7.0833E+00	1.1118E+01	1.2002E+01	2.7317E+00	1.0533E+01	5.3141E+00	1.2348E+01	1.0079E+00
F4	Mean	9.5124E+02	8.6721E+02	9.7728E+02	8.6139E+02	8.8065E+02	8.6317E+02	9.4197E+02	8.4649E+02
	Std	2.0323E+01	1.9903E+01	1.5175E+01	1.4093E+01	1.5579E+01	2.4242E+01	1.8185E+01	2.2566E+01
F5	Mean	3.9613E+03	1.6082E+03	3.9340E+03	1.1491E+03	2.3824E+03	1.1106E+03	2.7412E+03	9.8782E+02
	Std	6.2069E+02	2.9975E+02	4.7054E+02	3.2076E+02	2.7837E+02	3.2018E+02	6.1830E+02	7.2051E+01
F6	Mean	2.2708E+03	4.8236E+03	2.0072E+09	1.6381E+04	1.2036E+04	1.3246E+04	1.6047E+08	6.1906E+03
	Std	8.8172E+02	3.6237E+03	1.4667E+09	8.1059E+03	3.2956E+04	6.4208E+03	2.1748E+08	3.8166E+03
F7	Mean	2.2234E+03	2.1326E+03	2.2505E+03	2.0896E+03	2.1446E+03	2.0937E+03	2.1925E+03	2.0778E+03
	Std	7.6263E+01	5.2554E+01	4.7371E+01	4.2928E+01	2.6826E+01	4.8956E+01	7.0002E+01	2.9512E+01
F8	Mean	2.3496E+03	2.2908E+03	2.3440E+03	2.2394E+03	2.2470E+03	2.2537E+03	2.3536E+03	2.2333E+03
	Std	7.5287E+01	7.3512E+01	1.1916E+02	2.3540E+01	1.5166E+01	4.5677E+01	8.6181E+01	3.0313E+01
F9	Mean	2.4726E+03	2.5018E+03	3.0079E+03	2.4808E+03	2.5516E+03	2.4817E+03	2.7387E+03	2.4808E+03
	Std	2.7711E+01	1.7481E+01	1.6002E+02	3.9357E−02	3.9347E+01	6.9541E−01	9.0225E+01	1.1157E−09
F10	Mean	4.5285E+03	3.5971E+03	6.1164E+03	3.8215E+03	4.5215E+03	2.6965E+03	4.9643E+03	2.9219E+03
	Std	5.9652E+02	7.9996E+02	1.5845E+03	8.7856E+02	8.9723E+02	2.5978E+02	1.7090E+03	5.5181E+02
F11	Mean	2.7400E+03	3.1569E+03	8.5744E+03	2.9890E+03	3.1892E+03	2.9234E+03	6.9420E+03	2.9074E+03
	Std	1.5222E+02	3.8331E+02	6.5678E+02	1.5913E+02	3.5204E+02	6.1345E+01	1.2553E+03	6.9189E+01
F12	Mean	3.5861E+03	2.9940E+03	3.2414E+03	2.9605E+03	3.1368E+03	2.9764E+03	3.1859E+03	2.9729E+03
	Std	1.8476E+02	3.9662E+01	1.7137E+02	2.1140E+01	1.4377E+02	4.4805E+01	1.3799E+02	2.4334E+01

**Table 6:** Friedman test ranking summary.

Suites	CEC2017		CEC2022			
Dimensions	30		10		20	
**Algorithms**	M.R	T.R	M.R	T.R	M.R	T.R
MLPSO	4.87	5	5.50	6	5.17	5
MELGWO	4.30	4	3.92	3	4.08	4
MHWOA	7.70	8	7.75	8	7.67	8
ALA	2.80	2	2.17	2	2.83	2
HO	5.33	6	4.75	5	5.25	6
RIME	3.20	3	3.92	3	2.83	2
DOA	6.47	7	6.25	7	6.58	7
HSIDOA	1.33	1	1.75	1	1.58	1

**Table 7:** Evaluation metrics used for image segmentation.

Metric	Description	Reference
Peak Signal-to-Noise Ratio (PSNR)	Measures reconstruction quality based on pixel-level error	[41]
Structural Similarity (SSIM)	Evaluates structural similarity in terms of luminance, contrast, and structure	[10,42]
Feature Similarity (FSIM)	Assesses feature similarity using phase congruency and gradient information	[10,42]

## Data Availability

Data is contained within the article.
